# NCACO-score: An effective main-chain dependent scoring function for structure modeling

**DOI:** 10.1186/1471-2105-12-208

**Published:** 2011-05-26

**Authors:** Liqing Tian, Aiping Wu, Yang Cao, Xiaoxi Dong, Yun Hu, Taijiao Jiang

**Affiliations:** 1 National Laboratory of Biomacromolecules, Institute of Biophysics, Chinese Academy of Sciences, Beijing 100101, China; 2 Graduate School of the Chinese Academy of Sciences, Beijing 100080, China

## Abstract

**Background:**

Development of effective scoring functions is a critical component to the success of protein structure modeling. Previously, many efforts have been dedicated to the development of scoring functions. Despite these efforts, development of an effective scoring function that can achieve both good accuracy and fast speed still presents a grand challenge.

**Results:**

Based on a coarse-grained representation of a protein structure by using only four main-chain atoms: N, Cα, C and O, we develop a knowledge-based scoring function, called NCACO-score, that integrates different structural information to rapidly model protein structure from sequence. In testing on the Decoys'R'Us sets, we found that NCACO-score can effectively recognize native conformers from their decoys. Furthermore, we demonstrate that NCACO-score can effectively guide fragment assembly for protein structure prediction, which has achieved a good performance in building the structure models for hard targets from CASP8 in terms of both accuracy and speed.

**Conclusions:**

Although NCACO-score is developed based on a coarse-grained model, it is able to discriminate native conformers from decoy conformers with high accuracy. NCACO is a very effective scoring function for structure modeling.

## Background

A central stage at the protein structure modeling is to develop an effective energy function, also called potential or scoring function, which generally fall into two categories: physical-based and knowledge-based energy functions. Physical-based energy functions are derived from the laws of physics, which often use molecular mechanics method [1-3]. Whereas, knowledge-based energy functions are based on statistical analysis of experimentally determined protein structures, which provide an excellent shortcut towards a powerful energy function [4]. Compared to physical-based energy functions, knowledge-based energy functions have become more and more popular in protein structure prediction due to the relatively easy generation and manipulation of model structures and the lower computational cost. This can be seen from recent CASPs (Critical Assessment of Techniques for Protein Structure Prediction), in which the most successful prediction methods use knowledge-based energy functions [5,6]. Moreover, knowledge-based approaches have also been widely used in protein design [7], validation of experimentally determined protein structures [8,9] and protein-protein and protein-ligand interactions [10].

Knowledge-based energy functions can also be developed at different level of complexity depending on the level of structural details considered in the structural models. At the most detailed structural level, the knowledge-based energy functions require the structural information of all heavy atoms [4,11-19]. The all-atom knowledge-based energy functions can achieve good accuracy in structural analysis, but they typically incur intensive computation [20]. To reduce running time, many efforts have attempted to develop coarse-grained models with reduced structural representation [21-32]. The simplest coarse-grained model is to represent a residue as a point and thus a protein structure can be delineated as a simple C_α_-trace. The coarse-grained models at residue level indeed can significantly decrease the running time in structure modeling but they usually suffer unsatisfactory accuracy [21].

To balance the accuracy and computational time, the intermediate models between the atom-level and residue-level representations have also been developed [15,21,22,24]. In these models, the side chains are considered, which are usually simplified as C_β _atoms or the side chain center of mass. For example, in low resolution structure prediction, the Rosetta of Baker lab used a structural model represented by heavy main-chain atoms and pseudo side chain center of mass [33]. More recently, Makino and Itoh also developed a knowledge-based potential named DFMAC that requires the coordinates of main-chain atoms (N, C_α _and C) and pseudo C_β _atom [21]. Since the side chain conformations have been abstracted, these main-chain dependent models can achieve both relatively good accuracy and reasonable running time. Although these energy models have showed high ability of structure discrimination on known decoy data sets, few have demonstrated success in structure modeling, especially in de novo structure prediction. This is because de novo structure prediction requires high-performance scoring function not only with high accuracy of structure discrimination at low computational expense, but also with a smooth energy landscape which goes beyond the ability to distinguish the known decoy data sets. For example, DFMAC uses an orientation-dependent potential term between two C_α_-pseudo-C_β _vectors. Although the orientation-dependent potential showed a considerable improvement in distinguishing native structures and non-native (or decoy) structures, it relies on strict geometry features which are difficult to be satisfied due to the inaccuracy of pseudo atoms. This could significantly affect its use in structural modeling. Therefore, development of scoring functions for effective structure modeling still remains a challenging problem.

Here we also attempt to develop an effective knowledge-based scoring function based on main-chain dependent coarse-grained model that only requires the main-chain atoms (N C_α _C and O) coordinates per residue, which we called as NCACO-score. In developing NCACO-score, we considered an integration of four potential terms: contact-based pairwise atom-atom interactions, sequence-dependent local conformational propensities, solvation effects, and geometry propensities of pairwise triplet fragments on beta sheet. Although the first three terms follow the same form of other knowledge-based scoring functions [11,21], they are derived differently and carefully parameterized in the integration (see Methods). Moreover, we proposed a new potential term, the fourth term, to promote the formation of beta sheet during the structure modeling. The testing on 32 decoy sets from Decoys'R'Us database [34] shows that NCACO-score outperforms other coarse-grained potentials in discriminating native structures from their decoy structures. Remarkably, its average Z-score is much lower than those other coarse-grained potentials, indicating that NCACO-score has a larger energy gap between the native state and nonnative ones. This suggests its potential and effectiveness in structure prediction [13]. Furthermore, in an application of the scoring function to structure prediction using a simple fragment-based assembly procedure, we found that the structures modeled for the 14 hard targets of CASP8 achieved comparable accuracy with those predicted by Robetta [35]. The average running time of prediction for these targets was ~64.5 h of CPU time. This demonstrates the effectiveness of the NCACO-score in structure modeling. We believe that the scoring function could be very useful for protein structure modeling.

## Results

### Overview of NCACO-score

In NCACO-score, we consider two levels of coarse-grained structure representations. One is a five-bead model, in which a residue is represented as four main-chain atoms (N Cα C and O) and a pseudo side chain center of mass (except GLY). The pseudo Side chain Center of Mass (SCM) was determined according to the *φ/ψ *backbone torsion angles of the residue. We use the five-bead model to derive pairwise atom-atom contact potential (*E*_*con*_) and sequence-dependent local conformational potential (*E*_*trp*_). The other is a residue-level structure representation requiring Cα coordinates only. We use the residue-level model to derive solvation potential (*E*_*sol*_) and beta sheet geometry propensity potential (*E*_*beta*_). Therefore, our NCACO-score integrates the above four terms:(1)

The potential components considered in NCACO-score could reflect different aspects of protein folding principle. *E*_*con *_stands for the mean interaction potential between two atoms, which reflects the compatibility of the whole protein structure. This potential is the most widely used term in knowledge-based scoring function, and is even used alone in some pioneers' work [13,15,28,32]. *E*_*trp *_reflects the local conformational biases of secondary structure, which is local-sequence dependent. *E*_*sol *_stands for the solvation energy of protein, which reflects the hydrophobic effect of protein folding. We used a simple model based on Cα atom to derive the solvation energy by following Makino and Itoh's method [21]. To promote the formation of beta sheet, we introduced *E*_*beta *_to capture the geometrical features of beta sheet at residue level. The details of these four terms and their integration are described in Methods. Although it's difficult to construct orthogonal potential components for knowledge-based scoring function, we will show below that the integration of these four terms indeed improves structure discrimination ability, enabling us to develop an effective knowledge-based scoring function.

### Assessment of structure discrimination ability of NCACO-score and its individual terms

To gain insights into how well NCACO-score and its individual components discriminate native structures from non-native structures (also called decoys), we looked into their performance of structure discrimination on the Decoys'R'Us sets. 32 proteins from five decoy sets were selected: 1) 4state_reduced (seven proteins), 2) fisa (four proteins), 3) fisa_casp3 (three proteins), 4) lmds (ten proteins), and 5) lattice_ssfit (eight proteins).

Table 1 summarizes the discrimination power of NCACO-score and its four individual potential terms on the five decoy sets in terms of the ranks and Z-scores of native structures. As shown in the table, NCACO-score ranked the first the native structures of 23 proteins out of 32 proteins. Remarkably, the Z-scores of native structures were very low in most of the 32 proteins with average Z-score of -5.06, demonstrating the great structure discrimination ability of NCACO-score. Despite its overall good performance, NCACO-score could not effectively discriminate between the native and decoy structures for the two proteins (1fc2 in fisa set, and 1bba in lmds set) (z-score > 0), which we will explain below.

**Table 1:** Performance of single terms and the total scoring function of NCACO-score on Decoys'R'Us sets.

	**Size**^**a**^	***E***_***con***_		***E***_***trp***_		***E***_***sol***_		***E***_***beta***_		***E***_***tot***_	
4state_reduced											
1ctf	630	1^b^	-3.03^c^	1	-5.62	3	-1.92	3	-4.46	1	-5.48
1r69	676	2	-2.72	1	-4.94	29	-1.64	-^d^	-	1	-4.47
1sn3	660	1	-2.52	1	-9.58	175	-0.68	46	-1.51	1	-4.73
2cro	673	17	-1.99	1	-4.74	46	-1.42	-	-	1	-4.36
3icb	654	47	-1.42	1	-3.89	21	-1.48	59	-0.97	1	-3.33
4pti	686	9	-2.34	1	-9.51	55	-1.30	147	-0.62	1	-6.10
4rxn	677	27	-1.65	1	-5.17	74	-1.17	3	-3.74	1	-4.22
Average		14.86	-2.24	1.00	-6.21	57.57	-1.37	51.60	-2.26	1.00	-4.67
Fisa											
1fc2	501	9	-1.99	499	3.32	6	-1.92	-	-	461	1.68
1hdd-C	501	3	-3.08	375	0.61	1	-4.27	-	-	21	-1.55
2cro	501	13	-2.10	16	-1.74	56	-1.21	-	-	3	-2.43
4icb	500	1	-3.30	4	-2.28	2	-3.14	1	-42.13	1	-4.43
Average		6.50	-2.62	223.50	-0.02	16.25	-2.64	1.00	-42.13	121.50	-1.68
fisa_casp3											
1bg8-A	1200	377	-0.47	8	-2.23	850	0.55	-	-	44	-1.69
1bl0	972	818	1.02	1	-4.30	730	0.60	-	-	3	-2.46
1jwe	1407	387	-0.64	6	-2.66	343	-0.80	-	-	6	-2.33
Average		527.33	-0.03	5.00	-3.06	641.00	0.12	-	-	17.67	-2.16
lmds											
1b0n-B	498	4	-2.54	17	-1.99	136	-0.56	-	-	1	-2.56
1bba	501	294	0.26	498	1.69	348	0.43	-	-	497	2.02
1ctf	496	1	-2.58	1	-6.53	1	-2.82	223	-0.15	1	-6.28
1dtk	216	1	-2.42	86	-0.36	5	-1.75	110	0.16	8	-1.64
1fc2	501	71	-1.10	420	1.00	5	-2.45	-	-	113	-0.71
1igd	501	71	-1.03	1	-4.30	74	-1.07	9	-2.17	1	-4.38
1shf-A	437	35	-1.42	1	-6.85	101	-0.77	111	-0.57	1	-3.92
2cro	501	1	-6.13	2	-3.20	4	-2.45	-	-	1	-5.58
2ovo	348	37	-1.27	1	-8.53	26	-1.36	12	-2.44	1	-6.60
4pti	344	3	-2.54	1	-6.28	14	-1.78	245	0.81	1	-3.48
Average		51.80	-2.08	102.80	-3.54	71.40	-1.46	118.33	-0.73	62.50	-3.31
lattice_ssfit											
1beo	1998	61	-2.06	1	-19.51	1	-3.43	41	-1.76	1	-13.89
1ctf	1999	1	-4.03	1	-10.97	1	-3.48	1	-10.64	1	-13.75
1dkt-A	1995	82	-1.79	1	-7.28	64	-1.83	1	-12.29	1	-8.07
1fca	2001	72	-1.96	1	-8.82	65	-1.84	1	-11.71	1	-7.74
1nkl	1995	1	-3.86	1	-4.44	2	-3.28	-	-	1	-6.36
1pgb	1997	65	-1.98	1	-15.56	45	-1.99	1	-28.85	1	-16.66
1trl-A	1999	347	-0.97	1	-7.49	527	-0.63	-	-	1	-7.58
4icb	1998	525	-0.63	1	-8.75	2	-2.84	33	-1.56	1	-8.73
Average		144.25	-2.16	1.00	-10.35	88.38	-2.42	13.00	-11.14	1.00	-10.35

Summary											
Average		105.75	-2.01	61.00	-5.34	119.13	-1.68	58.17	-6.92	36.84	-5.06

^a ^The decoy structures with broken backbone or missing fragments were removed from our test.

^b ^The rank of native structure relative to decoy structures based on the calculated respective energies.

^c ^The Z-score of native structure in the decoy structures.

^d ^*E*_*beta *_is not evaluated due to no beta sheet in the protein.

For individual terms, *E*_*trp *_had the best performance, which ranked the first 21 of 32 native structures with average Z-score of -5.34, indicating that it contributed the most to NCACO-score. Although *E*_*con *_and *E*_*sol *_overall had lower ability of structure discrimination compared to *E*_*trp*_, they outperformed *E*_*trp *_in the fisa set. *E*_*beta*_, which is designed to capture the structural feature of beta sheet, indeed showed a good performance for most proteins containing beta sheet (average Z-score = -6.92).

From above, we can see that the performance of NCACO-score largely relies on the individual term *E*_*trp*_. Therefore, the extremely poor performance of *E*_*trp *_in discriminating the native and decoy structures of four proteins (1fc2 and 1hdd-C in fisa set, 1bba and 1fc2 in lmds set) could significantly affect the performance of NCACO-score in these four proteins. However, *E*_*con *_and *E*_*sol *_have better structure discrimination than *E*_*trp *_in these four proteins. As shown in Table 1, the integration of these two terms improves the performance NCACO-score, particularly for 1hdd-C in fisa set and 1fc2 in lmds set, in which integrated NCACO-score significantly outperforms *E*_*trp*_. This suggests the necessity of the integration of these different terms in developing an effective scoring function.

The decoy discrimination ability of NCACO-score was also rigorously evaluated using 4-fold cross-validations on the Decoys'R'Us sets (see additional file 1). As seen from the results of cross-validations, NCACO-score performed nearly equally well in both training sets and testing sets not only for the average rank of native structures (38.44 in training sets vs 36.96 in testing sets) but also for the Z-score of native structures (-5.10 in training sets vs -4.93 in testing sets). The close performance of our model in training sets and testing sets suggests the robustness and reliability of our model.

A good energy function should be able to discriminate native structure from near-native decoys [36]. However, Decoys'R'Us sets are not suitable for this test, because most of the decoys are far from native structures. Therefore, we further generated a new near-native decoy set by fragment replacement method (see additional file 2). This data set consists of 87 proteins with decoys of TM-scores between 0.6 and 0.9 compared to the native structure (see additional file 2). When NCACO-score was tested on this data set, the average Pearson correlation coefficient between energy and cRMSD for the 87 proteins was 0.64 (see additional file 2), demonstrating the reliability of NCACO-score in discrimination of near native structures.

### Comparison with other knowledge-based scoring functions

We compared the performance of NCACO-score on the 32 Decoys'R'Us sets with 6 state-of-the-art knowledge-based scoring functions: RAPDF [19], Atomic KBP [13], DFIRE-A [15], DFIRE-B [15], PC2CA [22], and DFMAC [21]. Table 2 shows comparison of the ranks and Z-scores of the native structures. RAPDF, Atomic KBP, and DFIRE-A need coordinates of all heavy atoms of main chain and side chain. DFIRE-B needs information of main chain and C_β_. PC2CA needs information of C_α _and C_β_. DFMAC needs information of the main-chain atoms (N, C_α_, and C). Like DFMAC, our scoring function NCACO-score needs coordinates of the main-chain atoms (N, C_α_, C, and O). The comparison results showed NCACO-score had the best discrimination performance on the 32 proteins in terms of both the average rank and the average Z-score. Among the four functions which used coarse-grained model (DFIRE-B, PC2CA, DFMAC, and NCACO-score), NCACO-score had the lowest average Z-score (-5.06), followed by DFMAC (-4.05), PC2CA (-3.48) and DFIRE-B (-3.32). For the average rank of native structure, NCACO-score also gave the best rank (36.84), followed by PC2CA (39.09) DFIRE-B (40.81) and DFMAC (47.53).

**Table 2:** Comparison of performance between NCACO-score and other potential functions on Decoys'R'Us sets.

		RAPDF		Atomic KBP		DFIRE-A		DFIRE-B		PC2CA			DFMAC			NCACO-score		
		
ID	**Size**^**a**^	**Rank**^**b**^	**Z-score**^**c**^	Rank	Z-score	Rank	Z-score	Rank	Z-score	Rank	Z-score	**C.C. **^**d**^	Rank	Z-score	C.C.	Rank	Z-score	C.C.
4state_reduced																		
1ctf	630	1	-3.26	1	-3.53	1	-3.86	1	-3.03	1	-3.4	0.59	1	-4.49	0.82	1	-5.48	0.73
1r69	676	1	-3.49	1	-3.76	1	-4.23	1	-2.95	1	-4	0.62	1	-3.34	0.82	1	-4.47	0.64
1sn3	660	1	-3.26	1	-3.5	1	-3.79	1	-3.4	1	-3.6	0.36	1	-3.14	0.55	1	-4.73	0.46
2cro	673	1	-2.93	1	-2.91	1	-3.29	2	-2.74	1	-3.2	0.69	1	-3.17	0.82	1	-4.36	0.59
3icb	654	1	-2.22	1	-2.41	4	-2.28	24	-1.68	1	-2.9	0.76	1	-2.01	0.86	1	-3.33	0.78
4pti	686	1	-3.12	1	-3.47	1	-3.62	1	-3.15	1	-3.1	0.40	1	-4.3	0.52	1	-6.1	0.39
4rxn	677	1	-2.79	1	-3.12	1	-3.33	19	-1.88	667	2.5	0.48	1	-2.9	0.67	1	-4.22	0.47
Average		1.00	-3.01	1.00	-3.24	1.43	-3.49	7.00	-2.69	96.14	-2.53	0.56	1.00	-3.34	0.72	1.00	-4.67	0.58
fisa																		
1fc2	501	497	2.74	413	1.05	254	-0.23	1	-2.76	1	-6.6	0.11	399	0.77	0.50	461	1.68	0.44
1hdd-C	501	17	-2	25	-1.78	1	-4.5	1	-6.76	1	-8.4	0.24	1	-4.81	0.39	21	-1.55	0.33
2cro	501	14	-1.93	24	-1.64	1	-6.33	1	-7.84	1	-7.3	0.17	1	-4.19	0.28	3	-2.43	0.26
4icb	500	1	-3.89	6	-2.46	1	-6.91	1	-8.47	1	-9.3	0.23	1	-5.1	0.25	1	-4.43	0.23
Average		132.25	-1.27	117.00	-1.21	64.25	-4.49	1.00	-6.46	1.00	-7.90	0.19	100.50	-3.33	0.36	121.50	-1.68	0.32
fisa_casp3																		
1bg8-A	1200	1	-4.39	2	-2.84	1	-5.35	1	-3.82	1	-4.5	0.26	14	-2.21	0.35	44	-1.69	0.17
1bl0	972	1	-3.19	215	-0.76	1	-4.5	3	-2.27	1	-3.1	-0.09	8	-2.17	0.30	3	-2.46	0.38
1jwe	1407	1	-4.69	4	-2.64	1	-6.26	1	-4.81	1	-5.6	0.10	1	-2.76	0.00	6	-2.33	-0.09
Average		1.00	-4.09	73.67	-2.08	1.00	-5.37	1.67	-3.63	1.00	-4.40	0.09	7.67	-2.38	0.22	17.67	-2.16	0.15
lmds																		
1b0n-B	498	359	0.45	74	-1.03	430	1.17	261	-0.03	1	-3.3	0.05	1	-2.82	0.07	1	-2.56	0.19
1bba	501	501	11.11	500	3.51	501	16.28	501	21.38	501	21.4	-0.23	501	4.38	0.18	497	2.02	0.24
1ctf	496	1	-2.84	1	-3.45	1	-3.54	1	-2.77	1	-3.4	0.31	1	-6.04	0.27	1	-6.28	0.12
1dtk	216	116	0.08	31	-1.16	1	-2.62	5	-2.46	2	-2.5	0.21	70	-0.38	0.04	8	-1.64	0.16
1fc2	501	501	7.75	501	8.86	501	5.72	441	1.22	53	-1.3	0.17	501	2.94	0.08	113	-0.71	0.01
1igd	501	1	-4.21	1	-4.16	1	-5.16	1	-4.69	1	-4	0.10	1	-7.21	0.25	1	-4.38	0.19
1shf-A	437	1	-5.15	2	-2.83	1	-6.68	1	-5.44	1	-5.3	0.11	1	-4.28	0.06	1	-3.92	0.02
2cro	501	416	0.96	175	-0.4	1	-4.7	1	-4.5	1	-7.7	0.13	1	-3.04	0.07	1	-5.58	0.06
2ovo	348	4	-2.76	1	-2.86	1	-3.21	27	-1.48	1	-3.2	0.11	1	-2.73	0.16	1	-6.6	0.15
4pti	344	157	-0.2	13	-1.75	1	-3.96	1	-3.47	1	-3.5	0.02	3	-2.57	0.10	1	-3.48	0.17
Average		205.70	0.52	129.90	-0.53	143.90	-0.67	124.00	-0.22	56.30	-1.28	0.10	108.10	-2.18	0.13	62.50	-3.31	0.13
lattice_ssfit																		
1beo	1998	1	-9.79	1	-9.47	1	-12.09	1	-7.95	1	-5.6	0.08	1	-8.37	0.04	1	-13.89	0.08
1ctf	1999	1	-6.99	1	-7.2	1	-10.05	1	-6.89	1	-6	0.03	1	-10.22	0.01	1	-13.75	0.10
1dkt-A	1995	1	-6.78	1	-6.78	1	-6.87	1	-4.92	1	-3.1	-0.01	1	-7.35	-0.05	1	-8.07	0.02
1fca	2001	1	-5.57	1	-3.36	1	-7.18	1	-5.3	1	-4.7	0.04	1	-9.31	0.01	1	-7.74	0.09
1nkl	1995	1	-8.33	1	-8.16	1	-9.29	1	-5.83	1	-4.1	0.01	1	-5.18	-0.09	1	-6.36	-0.14
1pgb	1997	1	-8.42	1	-6.86	1	-11.87	1	-9.64	1	-4.7	0.04	1	-13.65	0.14	1	-16.66	0.13
1trl-A	1999	1	-4.84	1	-5.58	1	-6.32	1	-3.73	1	-3.6	0.02	1	-4.09	-0.02	1	-7.58	0.01
4icb	1998	1	-6.68	1	-5.65	1	-7.81	1	-4.25	1	-4.4	0.00	1	-5.98	-0.02	1	-8.73	-0.02
Average		1.00	-7.18	1.00	-6.63	1.00	-8.94	1.00	-6.06	1.00	-4.53	0.03	1.00	-8.02	0.00	1.00	-10.35	0.03

Summary																		
Average		81.38	-2.83	62.59	-2.88	53.66	-4.27	40.81	-3.32	39.09	-3.48	0.19	47.53	-4.05	0.26	36.84	-5.06	0.23

^a ^The decoy structures with broken backbone or missing fragments were removed from our test.

^b ^The rank of native structure relative to decoy structures based on the calculated respective energies.

^c ^The Z-score of native structure in the decoy structures.

^d ^The Pearson correlation coefficient between energy and cRMSD.

For individual proteins, all the 6 functions showed similar trends with regard to their structure discriminating abilities. For example, they all discriminated 4state_reduced and lattice_ssfit sets with ease but were difficult to discriminate same proteins (e.g. 1fc2 in fisa set, 1bba and 1fc2 in lmds set). This phenomenon has been mentioned by other researchers before [15,37]. Zhou speculated that the failure of the scoring functions in 1bba could be that it was an atypical small protein without a significant hydrophobic core [15].

### Application to de novo protein structure prediction

Next, we sought to explore the performance of NCACO-score in structure modeling. Based on fragment assembly with three-residue fragments, we implemented a de novo structure prediction method that uses NCACO-score to guide the fragment assembly process (see Methods). To promote the formation of correct beta sheet, and prevent the formation of wrong beta sheet, the weight of *E*_*beta *_was changed periodically according to a sine function during the fragment assembly procedure. In order to avoid the excessive collisions between atoms during the assembly process, a simple energy term for punishing collision was added to the NCACO-score. The procedure iterated three times, and the predicted structures from the previous iteration were used as the initial structures of the next iteration.

To compare our prediction method with Robetta, one of the best existing protein structure prediction servers, we tested 14 CASP8 hard targets, on which Robetta used template-free modeling method. For each target, 1000 structure models were generated in our method, then clustered and 5 top models were selected by a centroid-based clustering. Table 3 shows the accuracies of the top 1 models predicted by our method by comparing to the models that Robetta predicted in CASP8 which are available at the Robetta web site. As shown in the table, our method had average prediction accuracy of TM-score 0.290, comparable to that by Robetta (0.287).

**Table 3:** Comparison of performance between our method and Robetta on 14 CASP8 hard targets.

			TM-score of Best Model	
			
ID	Length	Secondary Structure	Robetta (CASP8)	Our method
T0397-D1	70	7beta	0.25	0.277
T0460	111	3alpha 5beta	0.262	0.233
T0465	157	5alpha 8beta	0.243	0.309
T0466	128	8beta	0.326	0.172
T0468	109	1alpha 7beta	0.253	0.241
T0476	108	4alpha 6beta	0.279	0.241
T0480	55	3beta	0.208	0.217
T0482	120	3alpha 5beta	0.352	0.243
T0495-D2	65	1alpha 2beta	0.312	0.364
T0496-D1	110	3alpha 6beta	0.235	0.312
T0496-D2	68	2alpha	0.291	0.463
T0510-D3	44	1alpha 3beta	0.147	0.335
T0513-D2	77	2alpha 4beta	0.581	0.33
T0514	145	2alpha 10beta	0.283	0.322

Average			0.287	0.29

**Figure 1:**
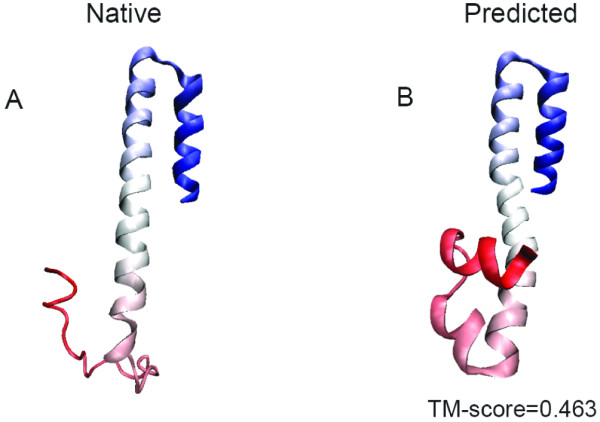
**Illustration of the predicted structure for T0496-D2**. The predicted model by NCACO-score (B) is compared with the experimental structure (A). Red to blue runs from the N to the C terminus. Structures are displayed by using VMD [48].

Although both our structure predictor and Robetta are based on fragment assembly, they have significantly different performance on modeling the structures. For example, for T0496-D2, our method correctly predicted its overall topology except for the coil part of its N-terminal (TM-score = 0.463) (comparing the predicted and native structure of T0496-D2 in Figure 1), which is much better than Robetta prediction (TM-score = 0.291). For the 14 targets, the Pearson's correlation coefficient between TM-scores of our predicted structures and those of Robetta-predicted structures is very low (0.085). The complementarity between the two methods underscores the importance of our work on the development of NCACO-score for structure modeling. Moreover, our method guided by NCACO-score has a moderate computational cost. For the 14 targets, the average CPU time was ~64.5 h on a 2.33 GHz Intel Xeon processor.

## Discussion

In this study, we have developed a knowledge-based scoring function named NCACO-score. NCACO-score integrates four different aspects of statistical structural features based on two coarse-grained models that only require coordinates of the heavy atoms of main-chain and pseudo side chain center of mass. We have demonstrated that NCACO-score can effectively discriminate native structures from their decoys, with a performance comparable to or even better than other state-of-the-art coarse-grained or all-atom knowledge-based statistical scoring functions. Moreover, NCACO-score can be used to guide fragment assembly for fast structure prediction, which can achieve a comparable accuracy to Robetta, one of the best structure modellers of similar kind.

In developing knowledge-based scoring functions, orientation-dependent structural features have been widely used [11,21,23,38]. For examples, DFMAC uses an orientation-dependent potential term between two C_α_-pseudo-C_β _vectors [21], and in OPUS-C_α_, the distance-dependent pairwise energy term and hydrogen bonding energy term are also orientation-dependent [23]. These orientation-dependent potentials are sensitive to geometrical features, causing the energy to be truncated abruptly at the cutoff. Therefore, these orientation-dependent potentials could have very rough energy landscape, which limit their applications to structural modeling. In NCACO-score, we exclude the orientation-dependent potentials that are sensitive to geometrical features, and only the coordinates of the heavy atoms of main-chain and pseudo side chain center of mass are needed, greatly simplifying structure representation without need to scan the side chain conformations. Moreover, unlike the directional hydrogen-bonding potentials for capturing the interaction of beta sheet, which depend on critical geometrical constraints for atoms, the novel term we developed to capture beta sheet was based on the propensity of geometrical features between beta-strand parings. As the geometrical features are defined at C_α _level in our model, the potential is less sensitive than typical directional hydrogen-bonding potential. We have shown that the novel beta sheet geometry propensity potential indeed had a good discrimination power for most proteins containing beta sheet.

Many potential terms were proposed and could be included in an effective knowledge-based scoring function. For example, Wu et al. added packing energy and three-body energy in their OPUS-C_α _potential [23], Makino and Itoh added the potential of ω dihedral angle in their DFMAC potential [21], and Fogolari et.al. added pseudo bond/angle/dihedral potentials in their PC2CA potential [22]. These potential terms are proved effective more or less in structure discrimination, which will be attempted in NCACO-score in our future work. The inclusion of more potential terms could improve the performance of structure discrimination, but this could render the energy landscape too rough and cause overfitting [39]. In developing NCACO-score, we have only considered four terms that reflect different aspects of protein folding principles. Indeed, integration of these four terms improves the performance of NCACO-score, although *E*_*trp *_makes a dominant contribution.

## Conclusions

NCACO-score is a knowledge-based scoring function that integrates four statistical structural features to effectively discriminate native structures from their decoys. Successful application of NCACO-score to structure modeling shows that NCACO-score could be a useful tool for structure modeling.

## Methods

### Nonhomologous structure database

6997 protein structures (less than 25% homology) with a resolution < 3.0Å and R-factor < 1.0 are obtained from PISCES server (May 20, 2010) [40], and are used to derive statistical potentials. For determining the distance cutoff of contact for any two types of atom, we need to get the minimal distance between any atom pair in protein structures (the detail of contact definition is below). So, a high-resolution structure database is used, including 2069 protein structures (less than 25% homology) with a resolution < 1.6Å and R-factor < 0.25 from PISCES server (May 20, 2010).

### Knowledge-based scoring function: NCACO-score

#### 1. Coarse-grained structure representations

**Figure 2:**
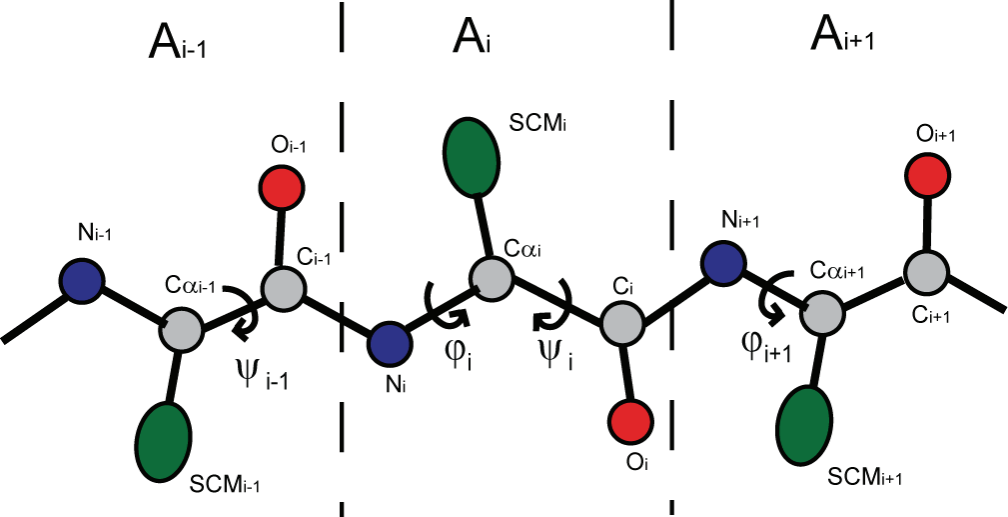
**Schematic representation of the backbone atoms (N Cα C O) and the side chain center of mass (SCM)**.

To calculate *E*_*con *_and *E*_*trp*_, NCACO-score uses a coarse-grained model of five beads, which includes 5 atoms (N Cα C O in backbone, and pseudo side chain center of mass) for representing a residue (Figure 2). For the *χ*_1_/*χ*_2 _torsion angles of side chain were dependent on its backbone *φ*/*ψ *torsion angles [41], the pseudo side chain center of mass was determined by its backbone *φ*/*ψ *torsion angles in our model, which is described as follows:

First, the side chain centers of mass (SCM) excluding H atoms for all residues of 6997 proteins are calculated. The *φ*/*ψ *torsion angles of backbone, the pseudo bond length (Cα-SCM), the pseudo bond angle (N-Cα-SCM), and the pseudo bond dihedral (N-C-Cα-SCM) are calculated for each residues. Second, the *φ*/*ψ *torsion angles (ranging from -180° to 180°) are divided into 36 bins with a width of 10°. The values of pseudo bond length, angle, and dihedral of the 19 amino acids (except GLY) according to each of the 36*36 bins are averaged on the 6997 proteins. Thus, a table containing the average pseudo bond length/angle/dihedral for each of all 19*36*36 pseudo SCM is obtained. Finally, given the four backbone atoms (N Cα C O) coordinates of a structure, the pseudo SCM information (bond length/angle/dihedral) for each residue is extracted from the table according to its amino acid type and *φ*/*ψ *torsion angles of backbone. The coordinate of pseudo SCM atom of each residue can be calculated by its backbone atoms (N Cα C O) coordinates and its pseudo SCM information through coordinates transformation.

To calculate *E*_*sol *_and *E*_*beta*_, we use residue-level coarse-grained model, in which a residue is represented as its Cα atom. Therefore, only the coordinates of Cα atoms are needed.

#### 2. Individual energy terms

##### The pairwise atom-atom contact potential

According to our five-bead coarse-grained model, there are four atoms (N Cα C O) for GLY and five atoms (N Cα C O SCM) for the other 19 amino acids, resulting in all 99 atom types. Two atoms A and B are defined to be in contact if their distance *d*_*AB *_is between *r*_*AB *_and λ*r_*AB*_, where *r*_*AB *_is the statistical shortest contact distance between atoms A and B observed in the 2069 high-resolution protein structures (*r*_*AB *_> 2.2Å to exclude the situation that two atoms could be covalently linked). *λ *= 1.9 is used in our model. Then, for each pair of atoms A and B, the number of contacts (*N*_*AB*_) and number of non-contacts  are counted in the 6997 proteins. By following the method of Shakhnovich lab [11], the contact potential uses the form of μ-potential defined as:(2)(3)

The value of μ = 0.987 is chosen to make the net interaction zero. *E*_*con *_for a protein is summed over the energies of all possible atom pairs in the protein.

##### The sequence-dependent local conformational potential

The Ramachandran map suggests that the distribution of *φ*/*ψ *torsion angles for a polypeptide is limited, and the distributions of *φ*/*ψ *torsion angles are different for different amino acids. We consider the local conformation potential based on three-residue fragment. Figure 2 shows a triplet fragment consisting of A_*i-*__1_, A_*i*_, A_*i*__+1_, where A_*i *_is the amino acid type of the *i*-th residue. The four dihedrals *ψ*_*i*-1 _*φ*_*i *_*ψ*_*i *_*φ*_*i *+ 1 _(range from -180° to 180°) are used to express the local conformation potential of a triplet fragment. Each dihedral is divided into 12 bins with bin width 30°. By following the method of Shakhnovich lab [11], the sequence-dependent local conformation potential is also expressed as a μ-potential:(4)(5)

where *N*_*j *_and  are the number of occurrence of the triplet A_*i-*__1_A_*i*_A_*i *__+ 1 _in the *j*-th bin and the total number of occurrence of the triplet A_*i-*__1_A_*i*_A_*i *__+ 1 _subtracted by *N*_*j *_in the 6997 proteins, respectively. The value of μ = 0.994 is chosen to make the net interaction zero. *E*_*trp *_for a protein is summed over the energies of all overlap triplet fragments in the protein.

##### The solvation potential

The solvation potential is derived at residue level by considering Cα atom only, which is similar to the SURR term used in DFMAC potential [21]. It is described as follows:(6)(7)(8)

where A_*i *_is the type of the *i*-th residue, and *E*_*ak *_is the potential for amino acid *a *with *k *contact residues. *N*_*ak *_is the number of observations for amino acid *a *with *k *contact residues in the 6997 proteins, and (the expectation of *N*_*ak*_) is estimated by equation 7. In the equation, *N*_*a *_is the total number of occurrence of amino acid *a*, *N*_*k *_is the total number of residues with *k *contact residues, and *N*_*total *_is the total number of residues in the database. In this procedure two residues are regarded to be in contact if the distance between their Cα atoms is less than 12.5Å. *E*_*sol *_for a protein is summed over the energies of all residues in the protein.

##### The beta sheet geometry propensity potential

**Figure 3:**
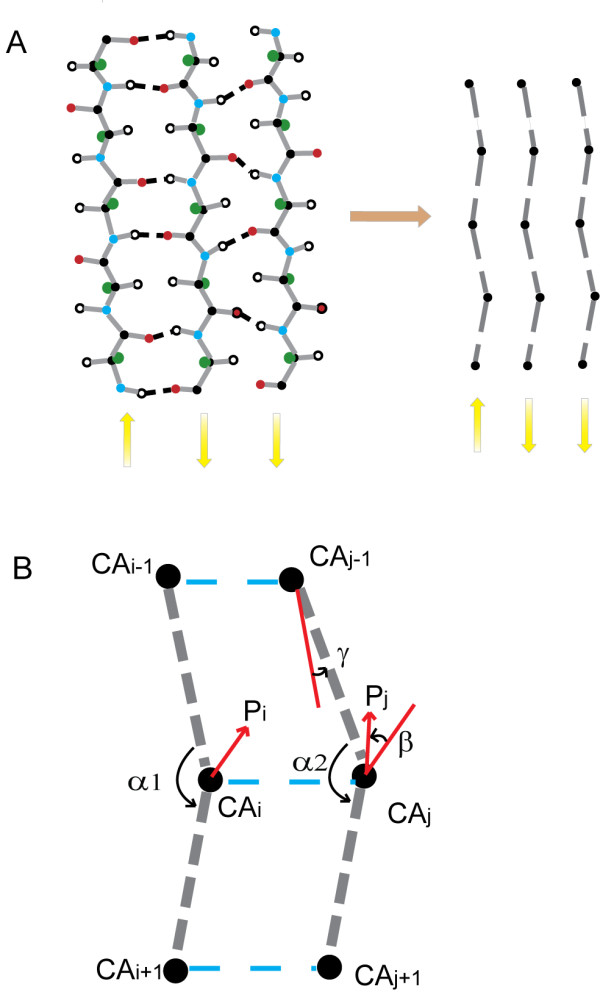
**Schematic illustration of the parameters used in the beta sheet geometry propensity potential**. A: Left is the representation of backbone atoms and side chain centroid atom of beta sheet. Right is the representation of Cα atoms. Using the representation of Cα atoms, the geometrical features of beta sheet is the same for both parallel and anti-parallel types. B: Four parameters *α*1 *α*2 *β γ *for a triplet pair in a beta sheet (parallel) are used to determine the geometrical tendency of beta sheet. *α*1 is the angle between two vector (CA_i-1_CA_i _and CA_i_CA_i+1_), and *α*2 is the angle between two vector (CA_j-1_CA_j _and CA_j_CA_j+1_). P_i _denotes the vector in the plane defined by three atoms (CA_i-1 _CA_i _CA_i + 1_) for residue CA_i_, and P_j _denotes the vector in the plane defined by three atoms (CA_j-1 _CA_j _CA_j + 1_) for residue CA_j_. *β *is the angle between P_i _and P_j_. *γ *is the angle between two vectors (CA_i-1_CA_i _and CA_j-1_CA_j_). The three blue dotted lines are represented the three distances that are used to define the tendency of forming beta sheet.

Hydrogen bond energy plays an important role in the formation of beta sheet. Thus far, most methods have attempted to calculate hydrogen-bond donor and acceptor explicitly to capture beta sheet [11,23,38], which are computationally intensive and cause the non-smooth truncation of energy at the geometric boundaries. To overcome the shortcoming, we used the geometrical features of beta sheet to guide the formation of beta sheet correctly based on a Cα level coarse-grained model. As shown in Figure 3A, both parallel and anti-parallel beta sheet have similar backbone geometrical feature at Cα level. Our model considers the pairing of three-residue fragments. First, we determine whether a pair of triplet fragments has a tendency to form beta sheet according to a simple criteria: all the three distances  and (see Figure 3B), are required to be less than a cutoff *d *for parallel beta sheet. While for anti-parallel beta sheet, the three distances are  and . The tendencies of both parallel and anti-parallel patterns are considered. If the both patterns are satisfied the criteria, we compare the average value of the three distances and choose the pattern that has a smaller average distance. Second, the four features *α*1 *α*2 *β *and *γ *(illustrated in Figure 3B) of a fragment pair are calculated for all fragment pairs that have tendencies to form beta sheet in the 6997 proteins. Then the four features are binned according to their range. The statistical results point that *α*1 and *α*2 fall into the range between 70° to 180° (varying slightly on different parameter *d *), which are separated into four bins: 0°~90°, 90°~120°, 120°~150°, and 150°~180°; *β *and *γ *fall into the range between 0° to 180°, which are separated into six bins: 0°~30°, 30°~60°, 60°~90°, 90°~120°, 120°~150°, and 150°~180°. In total, there are 4 × 4 × 6 × 6 = 576 bins for a fragment pair. Based on the Boltzmann law [42], the potential is derived as follows:(9)(10)(11)

where *N*_*α*__1__*α*__2__*βλ *_is the number of observations of all fragment pair in each bin of *α*1 *α*2 *β γ *in the database.  is the average number of observations for each bin. For parameter *d*, we tested its range from 5.0Å to 6.4Å and finally set it to 5.6Å which gives a better optimization result. 5.6Å is close to the average distance between backbones of beta sheet in the structure database. *E*_*beta *_for a protein is summed over the energies of all triplet fragment pairs that have tendencies to form beta sheet in the protein.

#### 3. Integration of the four terms into NCACO-score

The above four terms are linearly combined to form NCACO-score:(12)

where a, b, c are the relative weights of *E*_*trp*_, *E*_*sol *_and *E*_*beta *_respectively, which are parameterized on 32 Decoys'R'Us sets (see below).

#### 4. Optimization of weights and parameters

Weights of the equation 12 and parameters in the single potentials described above are optimized on 32 proteins of Decoys'R'Us sets (described above). The cost function for optimization is:(13)

where  is the average rank of native structure and  is the average Z-score for the proteins used for training. The optimization goal is to minimize the function value. After optimization on all 32 proteins, the NCACO-score is(14)

### Performance assessment

Two commonly used indices are used for quality measures: Rn, the rank of native structure relative to decoy structures based on the calculated energy; Z-score, a quantitative measure of energy bias for the native structure against decoy structures, which is defined as:(15)

 and *E*_*tot *_are the energies of the native and decoy structures, respectively,  and *σ*(*E*_*tot*_) are the average and standard deviation of energy of all decoys, respectively.

#### Development a de novo structure prediction method using fragment assembly guided by NCACO-score

We applied NCACO-score to structure modeling based on fragment assembly similar to that was used in Rosetta [33].

##### 1. Fragment templates library

In order to compare our prediction results with Robetta results in CASP8 fairly, the proteins in the database consisting of 6997 proteins that have more than 30% identity to the predicted target are excluded, which guarantees there are no homologous proteins for the predicted target. The list of fragment templates for the target proteins are generated as follows:

First, we obtained the secondary structure information for both target proteins and the template structures from the nonhomologous structure database. The secondary structures of target proteins are predicted using PSIPRED [43]. The secondary structures of the proteins in the nonhomologous structure database are identified using DSSP [44]: H, G, I for alpha helix, E for beta-strand, and the others for coil. Second, sequence profiles for each target sequence and each sequence of the structure database are constructed by three rounds of PSI-BLAST [45] with an e-value cutoff of 0.001. Finally, sequences of the target protein and the structure database are cut to overlapping triplet fragments. All triplet fragments of the structure database are scored with each triplet fragment of the target sequence using the following scoring function:(16)(17)(18)(19)

Where *F*_*query*_(*i, j*) and *P*_*template*_(*i, j*) are the frequency matrix of the query triplet and log-odds matrix of the template triplet for amino acid *j *at the *i*-th position, respectively. *q*_*i *_and *t*_*i *_are the secondary structure of query triplet and template triplet at the *i*-th position, respectively. After ranking, for each query triplet of the target protein, 25 triplets of top scores from the structure database are selected. The *φ*/*ψ*/*ω *torsion angles of template triplets are obtained as the fragment templates library.

##### 2. NCACO-score-guided structure modeling process

NCACO-score can be used to guide fragment assembly for protein structure prediction, which is detailed as follows:

*Step 1. *An extended backbone structure is initialized for the target sequence, in which all *φ*/*ψ*/*ω *torsion angles are 180°.

*Step 2. *Template triplets in the fragment templates library are randomly selected to replace the value of *φ*/*ψ*/*ω *torsion angles of the target sequence from N-term to C-term. Thus a random initial backbone structure is constructed, whose *φ*/*ψ*/*ω *torsion angles of backbone are all from the fragment templates library.

*Step 3*. A triplet in the target sequence is randomly selected as the target fragment, and is replaced by randomly selected template triplet. The energies of the structures are evaluated by NCACO-score with atom clash punishment and promotion of beta sheet (described below). Whether to reject or accept a replacement is based on the metropolis acceptance criteria: a replacement with lower energy is accepted, and a replacement with higher energy is occasionally accepted. The details are described below:(20)

where r is a random float number between 0 and 1. 35,000 replacements are implemented with a simulated annealing protocol. The temperature (T) is gradually decreased from 2000K to 300K. The initial factor is 1.0. In order to avoid local optima, if the times of continuous rejection (Nr) is more than 200, factor = 1.0+(Nr-200)/80.0; and if the factor is more than 11.0, the simulated annealing process is stopped.

*Step4. *The step2 and step3 are repeated for 1000 times to generate 1000 predicted structures for the target sequence.

*Step5. *Model selection. The 1000 predicted structures are clustered using a centroid-based clustering process by following the algorithm of SPICKER [46] with adaptation. The top five biggest clusters are selected as top five predicted structures. Different from SPICKER that uses RMSD to assess structural similarity for clustering, our implemented centroid-based clustering process uses TM-score, which is more sensitive than RMSD [47]. The initial TM-score cutoff is set to 0.7, the TM-score cutoff for clustering should be between 0.3 and 0.9, and the biggest cluster should includes 20% ~ 70% of the total number of structures.

The above procedure iterated for three times. In the second run, the top five predicted structures in the first run are used as initial structures. For each of the initial structures, the assembly procedure is implemented for 200 times, and it results in 200 predicted structures. The factor of metropolis acceptance criteria is different from that for the first run: if the times of continuous rejections Nr > 200, factor = 1.0+(Nr-200)/160.0; and if factor <6.0, the simulated annealing process is stopped. Similarly, the top five structures predicted in the second run are used as initial structures for the third run, and 200 times assembly procedures result in 200 predicted structures for each initial structure. In the third run, if the times of continuous rejection Nr > 200, factor = 1.0+(Nr-200)/320.0; and if the factor <3.5, the simulated annealing process is stopped. Finally, the structure with the lowest energy among the top five predicted structures from the third run is obtained as the best predicted model.

##### 3. Atom clash punishment and promotion of beta sheet

During the process of fragment assembly, atom clash happens frequently. Atoms A and B are regarded to have clash with each other if the distance *d*_*AB *_between them is less than the statistical shortest contact distance between them *r*_*AB *_(as described in the pairwise atom-atom contact potential part). Two measures are used to punish the atom clash. One is based on the number of all backbone atom pairs having clash (*N*_*bb*___*clash*_). If *N*_*bb*___*clash *_>*N*_*seq*_/3.0 (where *N*_*seq *_is the sequence length of the target protein), the structure is rejected. The other is to add a clash punishment term *E*_*clash *_= *N*_*clash *_to the NCACO-score. *N*_*clash *_is the total number of atom pairs in clash including the backbone atoms and the pseudo side chain center atoms.

As a long-range interaction, beta sheet is hard to form correctly during the fragment assembly process. To promote the correct formation of beta sheet, the weight of *E*_*beta *_varies by multiplying a periodic factor , where Tn is the step of random fragment replacement. Thus, the weight is increased during the first 500 steps. Then the weight is decreased and increased alternately.

## Authors' contributions

LT, AW and TJ designed the research. LT and AW carried out the study. LT and TJ drafted the manuscript. All authors contributed to the discussion and helped to draft the manuscript. All authors have read and approved the final manuscript.

## Supplementary Material

Additional file 1 **Cross-validation of decoy discrimination for NCACO-score on Decoys'R'Us sets**. In order to estimate the accuracy of the decoy discrimination for NCACO-score in practice, a 4-fold cross-validation was performed on the 32 proteins of Decoys'R'Us sets. These proteins were divided into four fold for average. The detail group information of the four sets including training set (24 proteins) and testing set (8 proteins) can be seen (Table S2 to Table S5), and the weights of NCACO-score for each sets were optimized by the training set. Click here for file

Additional file 2 **Test of NCACO-score in discriminating near-native decoys generated by fragment replacement**. In order to test whether NCACO-score is able to discriminate near-native decoys, we created a near-native decoy set by fragment replacement method. The decoy set contained 87 proteins, and each protein had 189 ~ 269 decoy structures with a TM-score of 0.6 ~ 0.9 relative to the native structure. Table S1 shows the performance of single terms and the total scoring function of NCACO-score on the near-native decoy set. Click here for file
