# Supplementary material for: NCACO-score: An effective main-chain dependent scoring function for structure modeling
Source: BMC Bioinformatics. 2011 May 26;12:208. doi: 10.1186/1471-2105-12-208 (PMC3123610; doi:10.1186/1471-2105-12-208)
Supplement: Additional file 1 — Cross-validation of decoy discrimination for NCACO-score on Decoys'R'Us sets. In order to estimate the accuracy of the decoy discrimination for NCACO-score in practice, a 4-fold cross-validation was performed on the 32 proteins of Decoys'R'Us sets. These proteins were divided into four fold for average. The detail group information of the four sets including training set (24 proteins) and testing set (8 proteins) can be seen (Table S2 to Table S5), and the weights of NCACO-score for each sets were optimized by the training set. [file 1471-2105-12-208-S1.DOC]

### Table S1: Summary of the 4-fold cross-validation results for NCACO-score on Decoys‘R’Us sets.

^a^ The average rank of native structure relative to decoy structures.

^b^ The average Z-score of native structure in the decoy structures.

|  |  | Training set | | Testing set | |
| --- | --- | --- | --- | --- | --- |
|  | Weights of NCACO after training | Rank^a^ | Z-score^b^ | Rank^a^ | Z-score^b^ |
| Set 1 | $E_{tot}=E_{con}+6.0E_{trp}+11.3E_{sol}+2.7E_{beta}$ | 28.33 | -5.44 | 69.63 | -4.59 |
| Set 2 | $E_{tot}=E_{con}+6.2E_{trp}+15.4E_{sol}+2.0E_{beta}$ | 47.92 | -4.55 | 2.13 | -6.25 |
| Set 3 | $E_{tot}=E_{con}+6.6E_{trp}+13.6E_{sol}+2.2E_{beta}$ | 44.96 | -5.26 | 17.25 | -4.88 |
| Set 4 | $E_{tot}=E_{con}+5.9E_{trp}+15.9E_{sol}+7.4E_{beta}$ | 26.63 | -5.15 | 64.75 | -4.00 |
| Average | | 36.96 | -5.10 | 38.44 | -4.93 |

### Table S2: Cross-validation results of Set 1 for NCACO-score on Decoys‘R’Us sets.

^a^ The decoy structures with broken backbone or fragment loss were removed from our test.

^b^ The rank of native structure relative to decoy structures based on the calculated respective energies.

^c^ The Z-score of native structure in the decoy structures.

^d^ $E_{beta}$ is not evaluated due to no beta sheet in the protein.

| ID | Size^a^ | $E_{con}$ | | $E_{trp}$ | | $E_{sol}$ | | $E_{beta}$ | | $E_{tot}$ | |
| --- | --- | --- | --- | --- | --- | --- | --- | --- | --- | --- | --- |
| *Training set* | | | | | | | | | | | |
| 4state_reduced | | | | | | | | | | | |
| 1r69 | 676 | 2^b^ | -2.72^c^ | 1 | -4.94 | 29 | -1.64 | -^d^ | - | 1 | -4.58 |
| 1sn3 | 660 | 1 | -2.52 | 1 | -9.58 | 175 | -0.68 | 46 | -1.51 | 1 | -5.10 |
| 2cro | 673 | 17 | -1.99 | 1 | -4.74 | 46 | -1.42 | - | - | 1 | -4.45 |
| 4pti | 686 | 9 | -2.34 | 1 | -9.51 | 55 | -1.30 | 147 | -0.62 | 1 | -6.58 |
| 4rxn | 677 | 27 | -1.65 | 1 | -5.17 | 74 | -1.17 | 3 | -3.74 | 1 | -4.36 |
| fisa | | | | | | | | | | | |
| 1fc2 | 501 | 9 | -1.99 | 499 | 3.32 | 6 | -1.92 | - | - | 476 | 1.93 |
| 2cro | 501 | 13 | -2.10 | 16 | -1.74 | 56 | -1.21 | - | - | 4 | -2.41 |
| 4icb | 500 | 1 | -3.30 | 4 | -2.28 | 2 | -3.14 | 1 | -42.13 | 1 | -4.32 |
| fisa_casp3 | | | | | | | | | | | |
| 1bg8-A | 1200 | 377 | -0.47 | 8 | -2.23 | 850 | 0.55 | - | - | 33 | -1.80 |
| 1jwe | 1407 | 387 | -0.64 | 6 | -2.66 | 343 | -0.80 | - | - | 5 | -2.44 |
| lmds | | | | | | | | | | | |
| 1b0n-B | 498 | 4 | -2.54 | 17 | -1.99 | 136 | -0.56 | - | - | 1 | -2.51 |
| 1ctf | 496 | 1 | -2.58 | 1 | -6.53 | 1 | -2.82 | 223 | -0.15 | 1 | -6.73 |
| 1dtk | 216 | 1 | -2.42 | 86 | -0.36 | 5 | -1.75 | 110 | 0.16 | 7 | -1.67 |
| 1fc2 | 501 | 71 | -1.10 | 420 | 1.00 | 5 | -2.45 | - | - | 137 | -0.51 |
| 1igd | 501 | 71 | -1.03 | 1 | -4.30 | 74 | -1.07 | 9 | -2.17 | 1 | -4.55 |
| 2cro | 501 | 1 | -6.13 | 2 | -3.20 | 4 | -2.45 | - | - | 1 | -5.58 |
| 2ovo | 348 | 37 | -1.27 | 1 | -8.53 | 26 | -1.36 | 12 | -2.44 | 1 | -7.08 |
| 4pti | 344 | 3 | -2.54 | 1 | -6.28 | 14 | -1.78 | 245 | 0.81 | 1 | -3.96 |
| lattice_ssfit | | | | | | | | | | | |
| 1ctf | 1999 | 1 | -4.03 | 1 | -10.97 | 1 | -3.48 | 1 | -10.64 | 1 | -13.80 |
| 1dkt-A | 1995 | 82 | -1.79 | 1 | -7.28 | 64 | -1.83 | 1 | -12.29 | 1 | -8.17 |
| 1fca | 2001 | 72 | -1.96 | 1 | -8.82 | 65 | -1.84 | 1 | -11.71 | 1 | -7.99 |
| 1pgb | 1997 | 65 | -1.98 | 1 | -15.56 | 45 | -1.99 | 1 | -28.85 | 1 | -16.87 |
| 1trl-A | 1999 | 347 | -0.97 | 1 | -7.49 | 527 | -0.63 | - | - | 1 | -7.86 |
| 4icb | 1998 | 525 | -0.63 | 1 | -8.75 | 2 | -2.84 | 33 | -1.56 | 1 | -9.07 |
| Summary | | | | | | | | | | | |
| Average |  | 88.50 | -2.11 | 44.71 | -5.36 | 108.54 | -1.65 | 59.50 | -8.34 | 28.33 | -5.44 |
| *Testing set* | | | | | | | | | | | |
| 4state_reduced | | | | | | | | | | | |
| 1ctf | 630 | 1 | -3.03 | 1 | -5.62 | 3 | -1.92 | 3 | -4.46 | 1 | -5.55 |
| 3icb | 654 | 47 | -1.42 | 1 | -3.89 | 21 | -1.48 | 59 | -0.97 | 1 | -3.42 |
| fisa | | | | | | | | | | | |
| 1hdd-C | 501 | 3 | -3.08 | 375 | 0.61 | 1 | -4.27 | - | - | 52 | -1.24 |
| fisa_casp3 | | | | | | | | | | | |
| 1bl0 | 972 | 818 | 1.02 | 1 | -4.30 | 730 | 0.60 | - | - | 3 | -2.73 |
| lmds | | | | | | | | | | | |
| 1bba | 501 | 294 | 0.26 | 498 | 1.69 | 348 | 0.43 | - | - | 497 | 1.99 |
| 1shf-A | 437 | 35 | -1.42 | 1 | -6.85 | 101 | -0.77 | 111 | -0.57 | 1 | -4.33 |
| lattice_ssfit | | | | | | | | | | | |
| 1beo | 1998 | 61 | -2.06 | 1 | -19.51 | 1 | -3.43 | 41 | -1.76 | 1 | -14.92 |
| 1nkl | 1995 | 1 | -3.86 | 1 | -4.44 | 2 | -3.28 | - | - | 1 | -6.51 |
| Summary | | | | | | | | | | | |
| Average |  | 157.50 | -1.70 | 109.88 | -5.29 | 150.88 | -1.76 | 53.5 | -1.94 | 69.63 | -4.59 |

### Table S3: Cross-validation results of Set 2 for NCACO-score on Decoys‘R’Us sets.

^a^ The decoy structures with broken backbone or fragment loss were removed from our test.

^b^ The rank of native structure relative to decoy structures based on the calculated respective energies.

^c^ The Z-score of native structure in the decoy structures.

^d^ $E_{beta}$ is not evaluated due to no beta sheet in the protein.

| ID | Size^a^ | $E_{con}$ | | $E_{trp}$ | | $E_{sol}$ | | $E_{beta}$ | | $E_{tot}$ | |
| --- | --- | --- | --- | --- | --- | --- | --- | --- | --- | --- | --- |
| *Training set* | | | | | | | | | | | |
| 4state_reduced | | | | | | | | | | | |
| 1ctf | 630 | 1^b^ | -3.03^c^ | 1 | -5.62 | 3 | -1.92 | 3 | -4.46 | 1 | -5.22 |
| 1sn3 | 660 | 1 | -2.52 | 1 | -9.58 | 175 | -0.68 | 46 | -1.51 | 1 | -4.64 |
| 2cro | 673 | 17 | -1.99 | 1 | -4.74 | 46 | -1.42 | -^d^ | - | 1 | -4.36 |
| 3icb | 654 | 47 | -1.42 | 1 | -3.89 | 21 | -1.48 | 59 | -0.97 | 1 | -3.27 |
| 4rxn | 677 | 27 | -1.65 | 1 | -5.17 | 74 | -1.17 | 3 | -3.74 | 1 | -3.92 |
| fisa | | | | | | | | | | | |
| 1fc2 | 501 | 9 | -1.99 | 499 | 3.32 | 6 | -1.92 | - | - | 459 | 1.64 |
| 1hdd-C | 501 | 3 | -3.08 | 375 | 0.61 | 1 | -4.27 | - | - | 17 | -1.63 |
| 4icb | 500 | 1 | -3.30 | 4 | -2.28 | 2 | -3.14 | 1 | -42.13 | 1 | -4.32 |
| fisa_casp3 | | | | | | | | | | | |
| 1bg8-A | 1200 | 377 | -0.47 | 8 | -2.23 | 850 | 0.55 | - | - | 49 | -1.65 |
| 1bl0 | 972 | 818 | 1.02 | 1 | -4.30 | 730 | 0.60 | - | - | 3 | -2.46 |
| lmds | | | | | | | | | | | |
| 1b0n-B | 498 | 4 | -2.54 | 17 | -1.99 | 136 | -0.56 | - | - | 1 | -2.51 |
| 1bba | 501 | 294 | 0.26 | 498 | 1.69 | 348 | 0.43 | - | - | 498 | 2.02 |
| 1ctf | 496 | 1 | -2.58 | 1 | -6.53 | 1 | -2.82 | 223 | -0.15 | 1 | -7.09 |
| 1fc2 | 501 | 71 | -1.10 | 420 | 1.00 | 5 | -2.45 | - | - | 106 | -0.77 |
| 1igd | 501 | 71 | -1.03 | 1 | -4.30 | 74 | -1.07 | 9 | -2.17 | 1 | -4.52 |
| 1shf-A | 437 | 35 | -1.42 | 1 | -6.85 | 101 | -0.77 | 111 | -0.57 | 1 | -4.31 |
| 2ovo | 348 | 37 | -1.27 | 1 | -8.53 | 26 | -1.36 | 12 | -2.44 | 1 | -6.55 |
| 4pti | 344 | 3 | -2.54 | 1 | -6.28 | 14 | -1.78 | 245 | 0.81 | 1 | -4.57 |
| lattice_ssfit | | | | | | | | | | | |
| 1beo | 1998 | 61 | -2.06 | 1 | -19.51 | 1 | -3.43 | 41 | -1.76 | 1 | -14.02 |
| 1dkt-A | 1995 | 82 | -1.79 | 1 | -7.28 | 64 | -1.83 | 1 | -12.29 | 1 | -7.13 |
| 1fca | 2001 | 72 | -1.96 | 1 | -8.82 | 65 | -1.84 | 1 | -11.71 | 1 | -7.38 |
| 1nkl | 1995 | 1 | -3.86 | 1 | -4.44 | 2 | -3.28 | - | - | 1 | -6.33 |
| 1trl-A | 1999 | 347 | -0.97 | 1 | -7.49 | 527 | -0.63 | - | - | 1 | -7.61 |
| 4icb | 1998 | 525 | -0.63 | 1 | -8.75 | 2 | -2.84 | 33 | -1.56 | 1 | -8.62 |
| Summary | | | | | | | | | | | |
| Average |  | 121.04 | -1.75 | 76.58 | -5.08 | 136.42 | -1.63 | 56.29 | -6.05 | 47.92 | -4.55 |
| *Testing set* | | | | | | | | | | | |
| 4state_reduced | | | | | | | | | | | |
| 1r69 | 676 | 2 | -2.72 | 1 | -4.94 | 29 | -1.64 | - | - | 1 | -4.44 |
| 4pti | 686 | 9 | -2.34 | 1 | -9.51 | 55 | -1.30 | 147 | -0.62 | 1 | -6.29 |
| fisa | | | | | | | | | | | |
| 2cro | 501 | 13 | -2.10 | 16 | -1.74 | 56 | -1.21 | - | - | 3 | -2.41 |
| fisa_casp3 | | | | | | | | | | | |
| 1jwe | 1407 | 387 | -0.64 | 6 | -2.66 | 343 | -0.80 | - | - | 6 | -2.33 |
| lmds | | | | | | | | | | | |
| 1dtk | 216 | 1 | -2.42 | 86 | -0.36 | 5 | -1.75 | 110 | 0.16 | 3 | -1.90 |
| 2cro | 501 | 1 | -6.13 | 2 | -3.20 | 4 | -2.45 | - | - | 1 | -5.41 |
| lattice_ssfit | | | | | | | | | | | |
| 1ctf | 1999 | 1 | -4.03 | 1 | -10.97 | 1 | -3.48 | 1 | -10.64 | 1 | -12.86 |
| 1pgb | 1997 | 65 | -1.98 | 1 | -15.56 | 45 | -1.99 | 1 | -28.85 | 1 | -14.33 |
| Summary | | | | | | | | | | | |
| Average |  | 59.88 | -2.80 | 14.25 | -6.12 | 67.25 | -1.83 | 64.75 | -9.99 | 2.13 | -6.25 |

### Table S4: Cross-validation results of Set 3 for NCACO-score on Decoys‘R’Us sets.

^a^ The decoy structures with broken backbone or fragment loss were removed from our test.

^b^ The rank of native structure relative to decoy structures based on the calculated respective energies.

^c^ The Z-score of native structure in the decoy structures.

^d^ $E_{beta}$ is not evaluated due to no beta sheet in the protein.

| ID | Size^a^ | $E_{con}$ | | $E_{trp}$ | | $E_{sol}$ | | $E_{beta}$ | | $E_{tot}$ | |
| --- | --- | --- | --- | --- | --- | --- | --- | --- | --- | --- | --- |
| *Training set* | | | | | | | | | | | |
| 4state_reduced | | | | | | | | | | | |
| 1ctf | 630 | 1^b^ | -3.03^c^ | 1 | -5.62 | 3 | -1.92 | 3 | -4.46 | 1 | -5.39 |
| 1r69 | 676 | 2 | -2.72 | 1 | -4.94 | 29 | -1.64 | -^d^ | - | 1 | -4.54 |
| 2cro | 673 | 17 | -1.99 | 1 | -4.74 | 46 | -1.42 | - | - | 1 | -4.44 |
| 3icb | 654 | 47 | -1.42 | 1 | -3.89 | 21 | -1.48 | 59 | -0.97 | 1 | -3.37 |
| 4pti | 686 | 9 | -2.34 | 1 | -9.51 | 55 | -1.30 | 147 | -0.62 | 1 | -6.65 |
| fisa | | | | | | | | | | | |
| 1fc2 | 501 | 9 | -1.99 | 499 | 3.32 | 6 | -1.92 | - | - | 475 | 1.87 |
| 1hdd-C | 501 | 3 | -3.08 | 375 | 0.61 | 1 | -4.27 | - | - | 40 | -1.33 |
| 2cro | 501 | 13 | -2.10 | 16 | -1.74 | 56 | -1.21 | - | - | 3 | -2.39 |
| fisa_casp3 | | | | | | | | | | | |
| 1bg8-A | 1200 | 377 | -0.47 | 8 | -2.23 | 850 | 0.55 | - | - | 35 | -1.76 |
| 1bl0 | 972 | 818 | 1.02 | 1 | -4.30 | 730 | 0.60 | - | - | 3 | -2.71 |
| 1jwe | 1407 | 387 | -0.64 | 6 | -2.66 | 343 | -0.80 | - | - | 5 | -2.43 |
| lmds | | | | | | | | | | | |
| 1bba | 501 | 294 | 0.26 | 498 | 1.69 | 348 | 0.43 | - | - | 498 | 2.00 |
| 1ctf | 496 | 1 | -2.58 | 1 | -6.53 | 1 | -2.82 | 223 | -0.15 | 1 | -7.19 |
| 1dtk | 216 | 1 | -2.42 | 86 | -0.36 | 5 | -1.75 | 110 | 0.16 | 4 | -1.80 |
| 1igd | 501 | 71 | -1.03 | 1 | -4.30 | 74 | -1.07 | 9 | -2.17 | 1 | -4.62 |
| 1shf-A | 437 | 35 | -1.42 | 1 | -6.85 | 101 | -0.77 | 111 | -0.57 | 1 | -4.53 |
| 2cro | 501 | 1 | -6.13 | 2 | -3.20 | 4 | -2.45 | - | - | 1 | -5.46 |
| 4pti | 344 | 3 | -2.54 | 1 | -6.28 | 14 | -1.78 | 245 | 0.81 | 1 | -4.55 |
| lattice_ssfit | | | | | | | | | | | |
| 1beo | 1998 | 61 | -2.06 | 1 | -19.51 | 1 | -3.43 | 41 | -1.76 | 1 | -14.94 |
| 1ctf | 1999 | 1 | -4.03 | 1 | -10.97 | 1 | -3.48 | 1 | -10.64 | 1 | -13.27 |
| 1fca | 2001 | 72 | -1.96 | 1 | -8.82 | 65 | -1.84 | 1 | -11.71 | 1 | -7.81 |
| 1nkl | 1995 | 1 | -3.86 | 1 | -4.44 | 2 | -3.28 | - | - | 1 | -6.47 |
| 1pgb | 1997 | 65 | -1.98 | 1 | -15.56 | 45 | -1.99 | 1 | -28.85 | 1 | -15.57 |
| 4icb | 1998 | 525 | -0.63 | 1 | -8.75 | 2 | -2.84 | 33 | -1.56 | 1 | -8.95 |
| Summary | | | | | | | | | | | |
| Average |  | 117.25 | -2.05 | 62.75 | -5.40 | 116.79 | -1.74 | 75.69 | -4.81 | 44.96 | -5.26 |
| *Testing set* | | | | | | | | | | | |
| 4state_reduced | | | | | | | | | | | |
| 1sn3 | 660 | 1 | -2.52 | 1 | -9.58 | 175 | -0.68 | 46 | -1.51 | 1 | -5.01 |
| 4rxn | 677 | 27 | -1.65 | 1 | -5.17 | 74 | -1.17 | 3 | -3.74 | 1 | -4.17 |
| fisa | | | | | | | | | | | |
| 4icb | 500 | 1 | -3.30 | 4 | -2.28 | 2 | -3.14 | 1 | -42.13 | 1 | -4.28 |
| lmds | | | | | | | | | | | |
| 1b0n-B | 498 | 4 | -2.54 | 17 | -1.99 | 136 | -0.56 | - | - | 1 | -2.48 |
| 1fc2 | 501 | 71 | -1.10 | 420 | 1.00 | 5 | -2.45 | - | - | 131 | -0.57 |
| 2ovo | 348 | 37 | -1.27 | 1 | -8.53 | 26 | -1.36 | 12 | -2.44 | 1 | -7.02 |
| lattice_ssfit | | | | | | | | | | | |
| 1dkt-A | 1995 | 82 | -1.79 | 1 | -7.28 | 64 | -1.83 | 1 | -12.29 | 1 | -7.64 |
| 1trl-A | 1999 | 347 | -0.97 | 1 | -7.49 | 527 | -0.63 | - | - | 1 | -7.85 |
| Summary | | | | | | | | | | | |
| Average |  | 71.25 | -1.89 | 55.75 | -5.16 | 126.13 | -1.48 | 12.6 | -12.42 | 17.25 | -4.88 |

### Table S5: Cross-validation results of Set 4 for NCACO-score on Decoys‘R’Us sets.

^a^ The decoy structures with broken backbone or fragment loss were removed from our test.

^b^ The rank of native structure relative to decoy structures based on the calculated respective energies.

^c^ The Z-score of native structure in the decoy structures.

^d^ $E_{beta}$ is not evaluated due to no beta sheet in the protein.

| ID | Size^a^ | $E_{con}$ | | $E_{trp}$ | | $E_{sol}$ | | $E_{beta}$ | | $E_{tot}$ | |
| --- | --- | --- | --- | --- | --- | --- | --- | --- | --- | --- | --- |
| *Training set* | | | | | | | | | | | |
| 4state_reduced | | | | | | | | | | | |
| 1ctf | 630 | 1^b^ | -3.03^c^ | 1 | -5.62 | 3 | -1.92 | 3 | -4.46 | 1 | -5.35 |
| 1r69 | 676 | 2 | -2.72 | 1 | -4.94 | 29 | -1.64 | -^d^ | - | 1 | -4.36 |
| 1sn3 | 660 | 1 | -2.52 | 1 | -9.58 | 175 | -0.68 | 46 | -1.51 | 1 | -4.49 |
| 3icb | 654 | 47 | -1.42 | 1 | -3.89 | 21 | -1.48 | 59 | -0.97 | 1 | -3.25 |
| 4pti | 686 | 9 | -2.34 | 1 | -9.51 | 55 | -1.30 | 147 | -0.62 | 1 | -5.85 |
| 4rxn | 677 | 27 | -1.65 | 1 | -5.17 | 74 | -1.17 | 3 | -3.74 | 1 | -4.08 |
| fisa | | | | | | | | | | | |
| 1hdd-C | 501 | 3 | -3.08 | 375 | 0.61 | 1 | -4.27 | - | - | 11 | -1.78 |
| 2cro | 501 | 13 | -2.10 | 16 | -1.74 | 56 | -1.21 | - | - | 3 | -2.41 |
| 4icb | 500 | 1 | -3.30 | 4 | -2.28 | 2 | -3.14 | 1 | -42.13 | 1 | -4.44 |
| fisa_casp3 | | | | | | | | | | | |
| 1bl0 | 972 | 818 | 1.02 | 1 | -4.30 | 730 | 0.60 | - | - | 4 | -2.34 |
| 1jwe | 1407 | 387 | -0.64 | 6 | -2.66 | 343 | -0.80 | - | - | 6 | -2.28 |
| lmds | | | | | | | | | | | |
| 1b0n-B | 498 | 4 | -2.54 | 17 | -1.99 | 136 | -0.56 | - | - | 1 | -2.52 |
| 1bba | 501 | 294 | 0.26 | 498 | 1.69 | 348 | 0.43 | - | - | 498 | 2.04 |
| 1dtk | 216 | 1 | -2.42 | 86 | -0.36 | 5 | -1.75 | 110 | 0.16 | 8 | -1.61 |
| 1fc2 | 501 | 71 | -1.10 | 420 | 1.00 | 5 | -2.45 | - | - | 91 | -0.87 |
| 1shf-A | 437 | 35 | -1.42 | 1 | -6.85 | 101 | -0.77 | 111 | -0.57 | 2 | -3.74 |
| 2cro | 501 | 1 | -6.13 | 2 | -3.20 | 4 | -2.45 | - | - | 1 | -5.34 |
| 2ovo | 348 | 37 | -1.27 | 1 | -8.53 | 26 | -1.36 | 12 | -2.44 | 1 | -6.42 |
| lattice_ssfit | | | | | | | | | | | |
| 1beo | 1998 | 61 | -2.06 | 1 | -19.51 | 1 | -3.43 | 41 | -1.76 | 1 | -13.54 |
| 1ctf | 1999 | 1 | -4.03 | 1 | -10.97 | 1 | -3.48 | 1 | -10.64 | 1 | -13.32 |
| 1dkt-A | 1995 | 82 | -1.79 | 1 | -7.28 | 64 | -1.83 | 1 | -12.29 | 1 | -7.87 |
| 1nkl | 1995 | 1 | -3.86 | 1 | -4.44 | 2 | -3.28 | - | - | 1 | -6.19 |
| 1pgb | 1997 | 65 | -1.98 | 1 | -15.56 | 45 | -1.99 | 1 | -28.85 | 1 | -16.27 |
| 1trl-A | 1999 | 347 | -0.97 | 1 | -7.49 | 527 | -0.63 | - | - | 1 | -7.39 |
| Summary | | | | | | | | | | | |
| Average |  | 96.21 | -2.13 | 59.96 | -5.52 | 114.75 | -1.69 | 41.23 | -8.45 | 26.63 | -5.15 |
| *Testing set* | | | | | | | | | | | |
| 4state_reduced | | | | | | | | | | | |
| 2cro | 673 | 17 | -1.99 | 1 | -4.74 | 46 | -1.42 | - | - | 1 | -4.29 |
| fisa | | | | | | | | | | | |
| 1fc2 | 501 | 9 | -1.99 | 499 | 3.32 | 6 | -1.92 | - | - | 454 | 1.52 |
| fisa_casp3 | | | | | | | | | | | |
| 1bg8-A | 1200 | 377 | -0.47 | 8 | -2.23 | 850 | 0.55 | - | - | 58 | -1.59 |
| lmds | | | | | | | | | | | |
| 1ctf | 496 | 1 | -2.58 | 1 | -6.53 | 1 | -2.82 | 223 | -0.15 | 1 | -3.95 |
| 1igd | 501 | 71 | -1.03 | 1 | -4.30 | 74 | -1.07 | 9 | -2.17 | 1 | -4.30 |
| 4pti | 344 | 3 | -2.54 | 1 | -6.28 | 14 | -1.78 | 245 | 0.81 | 1 | -3.34 |
| lattice_ssfit | | | | | | | | | | | |
| 1fca | 2001 | 72 | -1.96 | 1 | -8.82 | 65 | -1.84 | 1 | -11.71 | 1 | -7.68 |
| 4icb | 1998 | 525 | -0.63 | 1 | -8.75 | 2 | -2.84 | 33 | -1.56 | 1 | -8.37 |
| Summary | | | | | | | | | | | |
| Average |  | 134.38 | -1.65 | 64.13 | -4.79 | 132.25 | -1.64 | 102.2 | -2.96 | 64.75 | -4.00 |
