# Supplementary material for: NCACO-score: An effective main-chain dependent scoring function for structure modeling
Source: BMC Bioinformatics. 2011 May 26;12:208. doi: 10.1186/1471-2105-12-208 (PMC3123610; doi:10.1186/1471-2105-12-208)
Supplement: Additional file 2 — Test of NCACO-score in discriminating near-native decoys generated by fragment replacement. In order to test whether NCACO-score is able to discriminate near-native decoys, we created a near-native decoy set by fragment replacement method. The decoy set contained 87 proteins, and each protein had 189 ~ 269 decoy structures with a TM-score of 0.6 ~ 0.9 relative to the native structure. Table S1 shows the performance of single terms and the total scoring function of NCACO-score on the near-native decoy set. [file 1471-2105-12-208-S2.DOC]

**1 Generation of a near-native decoy set by fragment replacement.**

The procedure to generate near-native decoy set by fragment replacement is described as follows:

**Selection of target proteins**

100 proteins (50~300 residues) were selected from nonhomologous structure database (6997 proteins, see main text) randomly. The proteins with very simple structures, such as a protein consisting of only two alpha-helices linked by a loop, may not be suitable for generation of sufficient decoys, and thus were excluded. As result, 13 proteins were excluded and 87 proteins were selected as target proteins for generation of decoys.

**Preparation of fragment templates library for target proteins**

The 6997 proteins were separated into two sets: 87 target proteins for creating near-native decoy set, and the other 6910 proteins used for creating fragment templates (denoted as template proteins). The fragment templates libraries for target proteins were generated using the following steps:

1. The secondary structures of all the target and template proteins were identified using DSSP: H, G, I for alpha helix, E for beta-strand, and the others for coil.
2. The sequence profiles for all the target and template proteins were constructed by three rounds of PSI-BLAST with an e-value cutoff of 0.001.
3. Sequences of target proteins and the database proteins were cut to overlapping triplet fragments.
4. Each target triplet fragment is scored with all template triplet fragments that have the same secondary structures as the target triplet fragment. The score function is given as follows:

$S(query, template)=\sum_{i=1}^{3} \sum_{j=1}^{20} F_{query}(i,j)P_{template}(i,j)$ (1)

where $F_{query}(i,j)$ and $P_{template}(i,j)$ are the frequency matrix of the query triplet and log-odds matrix of the template triplet for amino acid *j* at the *i*-th position, respectively.

5. For each query triplet fragment of the target protein, 50 template triplets of top scores were selected. The torsion angles of template triplets were obtained as the fragment templates library for the given query triplet fragment.

**Generation of near-native decoy set using a fragment replacement procedure**

To generate a near-native decoy set, the fragment replacement starts from a native structure. In each operation, a triplet in the target sequence is randomly selected and replaced by a randomly selected template triplet. If the TM-score of the structure with replaced fragment compared to the native structure is lower than the given cutoff, cutoff1 (set 0.85 at the beginning), reject the replacement and try another replacement. If the TM-score is higher than cutoff1+0.05, accept the replacement and try another replacement until the TM-score is between cutoff1 and cutoff1+0.05. If the TM-score is between cutoff1 and cutoff1+0.05, output the structure as a decoy structure. Then, based on the decoy structure, we follow the above process to generate a new decoy structure by decreasing cutoff1 by 0.05 until 6 decoys are obtained. In this way, each decoy with lower TM-score is generated based on the one with higher TM-score. Therefore, the TM-scores of the six decoys are 0.85~0.9, 0.8~0.85 0.75~0.8, 0.7~0.75, 0.65~0.7, 0.6~0.7, respectively. This process was repeated 150 times and in total 900 decoys are created, whose TM-scores are nearly uniformly distributed from 0.6 to 0.9.

A filtering process is applied to the 900 decoys by using the following two steps:

Step 1. If the structures of two decoys are too similar (TM-score>0.9), remove one of them. Make sure the TM-score of any two decoys is lower than 0.9.

Step 2. Select one decoy in every 0.001 interval of TM-score from 0.6 to 0.9, which results in the decoys with uniform distribution of TM-scores from 0.6 to 0.9.

The final decoy set for the 87 proteins can be downloaded from <http://jianglab.ibp.ac.cn/lims/ncaco/ncaco.html>.

**2 Testing the performance of discriminating near-native decoys for NCACO-score.**

### Table S1: Performance of single terms and the total scoring function of NCACO-score on the near-native decoy set.

^a^ The number of decoy structures.

^b^ The rank of native structure relative to decoy structures based on the calculated respective energies.

^c^ The Z-score of native structure in the decoy structures.

^d^ The Pearson correlation coefficient between energy and cRMSD.

^e^ The loss of GDT_TS for the top-ranked model compared to the best model in the decoy set.

^f^$E_{beta}$ is not evaluated due to no beta sheet in the protein.

|  |  | $E_{con}$ | | | $E_{trp}$ | | | $E_{sol}$ | | | $E_{beta}$ | | | $E_{tot}$ | | | |
| --- | --- | --- | --- | --- | --- | --- | --- | --- | --- | --- | --- | --- | --- | --- | --- | --- | --- |
| ID | Size ^a^ | Rank ^b^ | Z-score ^c^ | C.C. ^d^ | Rank | Z-score | C.C. | Rank | Z-score | C.C. | Rank | Z-score | C.C. | Rank | Z-score | C.C. | GDT_TS loss ^e^ |
| 1AMFA | 256 | 1 | 0.77 | -2.54 | 41 | 0.39 | -1.00 | 1 | 0.73 | -3.83 | 1 | 0.64 | -2.78 | 1 | 0.78 | -3.19 | 0.05 |
| 1AYOA | 251 | 1 | 0.60 | -3.63 | 80 | 0.23 | -0.60 | 1 | 0.56 | -2.64 | 1 | 0.50 | -3.22 | 1 | 0.58 | -3.20 | 0.01 |
| 1B5EA | 235 | 1 | 0.76 | -2.64 | 77 | 0.15 | -0.55 | 2 | 0.75 | -2.67 | 2 | 0.55 | -2.65 | 2 | 0.72 | -2.82 | 0.06 |
| 1ETEA | 253 | 1 | 0.75 | -3.32 | 77 | 0.27 | -0.47 | 1 | 0.76 | -3.27 | 11 | -0.04 | -1.99 | 1 | 0.76 | -3.35 | 0.02 |
| 1H72C | 252 | 1 | 0.77 | -3.05 | 91 | 0.19 | -0.32 | 2 | 0.72 | -2.44 | 1 | 0.67 | -2.37 | 1 | 0.78 | -2.79 | 0.12 |
| 1HLMA | 241 | 34 | 0.48 | -1.11 | 225 | -0.28 | 1.40 | 7 | 0.63 | -1.91 | -^f^ | - | - | 36 | 0.47 | -1.06 | 0.24 |
| 1JBOA | 234 | 47 | 0.52 | -0.86 | 52 | 0.28 | -0.84 | 21 | 0.51 | -1.23 | - | - | - | 23 | 0.54 | -1.21 | 0.02 |
| 1JOSA | 218 | 16 | 0.58 | -1.61 | 58 | 0.17 | -0.70 | 6 | 0.56 | -1.95 | 2 | 0.27 | -2.75 | 4 | 0.59 | -2.37 | 0.01 |
| 1K0MA | 227 | 5 | 0.59 | -2.16 | 13 | 0.47 | -1.32 | 2 | 0.69 | -2.08 | 6 | 0.36 | -2.24 | 1 | 0.68 | -2.37 | 0.01 |
| 1LKKA | 253 | 3 | 0.61 | -2.23 | 104 | 0.32 | -0.34 | 1 | 0.64 | -2.30 | 1 | 0.26 | -1.81 | 1 | 0.65 | -2.36 | 0.05 |
| 1LR5A | 258 | 4 | 0.64 | -2.09 | 77 | 0.07 | -0.51 | 3 | 0.63 | -2.52 | 4 | 0.36 | -1.42 | 1 | 0.56 | -2.14 | 0.00 |
| 1MKKA | 232 | 1 | 0.64 | -3.85 | 43 | 0.04 | -0.90 | 1 | 0.55 | -3.28 | 1 | 0.64 | -3.21 | 1 | 0.63 | -3.66 | 0.00 |
| 1MQOA | 245 | 4 | 0.67 | -2.37 | 53 | 0.40 | -0.79 | 1 | 0.66 | -2.20 | 1 | 0.51 | -2.37 | 1 | 0.68 | -2.50 | 0.01 |
| 1MXIA | 247 | 2 | 0.54 | -2.52 | 49 | 0.16 | -0.90 | 1 | 0.61 | -2.25 | 1 | 0.16 | -2.54 | 1 | 0.51 | -2.79 | 0.04 |
| 1NZYA | 229 | 5 | 0.48 | -2.18 | 34 | 0.34 | -0.99 | 19 | 0.49 | -1.36 | 3 | 0.40 | -2.27 | 3 | 0.53 | -2.09 | 0.12 |
| 1OH0A | 240 | 6 | 0.64 | -2.39 | 19 | 0.43 | -1.31 | 2 | 0.50 | -2.00 | 1 | 0.56 | -2.66 | 1 | 0.70 | -2.87 | 0.03 |
| 1OO0B | 250 | 16 | 0.70 | -1.80 | 115 | 0.09 | -0.12 | 9 | 0.63 | -1.65 | 4 | 0.40 | -2.19 | 4 | 0.64 | -2.01 | 0.01 |
| 1OU8A | 250 | 3 | 0.67 | -1.89 | 24 | 0.23 | -1.25 | 2 | 0.65 | -1.95 | 13 | 0.44 | -1.31 | 2 | 0.64 | -2.01 | 0.01 |
| 1P3CA | 249 | 1 | 0.79 | -2.45 | 9 | 0.43 | -1.71 | 1 | 0.64 | -2.18 | 4 | 0.56 | -1.93 | 1 | 0.71 | -2.42 | 0.02 |
| 1Q0RA | 255 | 1 | 0.68 | -2.66 | 34 | 0.45 | -1.06 | 1 | 0.68 | -2.34 | 1 | 0.39 | -2.85 | 1 | 0.65 | -2.73 | 0.07 |
| 1QSOA | 237 | 24 | 0.58 | -1.42 | 136 | -0.06 | 0.19 | 10 | 0.71 | -1.57 | 2 | 0.21 | -1.63 | 4 | 0.64 | -1.82 | 0.03 |
| 1R8SA | 244 | 2 | 0.57 | -2.49 | 26 | 0.39 | -1.22 | 2 | 0.59 | -2.32 | 1 | 0.19 | -2.78 | 1 | 0.54 | -2.76 | 0.11 |
| 1RYP1 | 260 | 1 | 0.63 | -2.98 | 49 | 0.24 | -0.91 | 2 | 0.68 | -2.71 | 2 | 0.38 | -2.84 | 1 | 0.64 | -3.19 | 0.05 |
| 1SEFA | 246 | 2 | 0.76 | -2.83 | 40 | 0.39 | -0.97 | 2 | 0.61 | -2.44 | 1 | 0.62 | -2.62 | 1 | 0.74 | -2.90 | 0.09 |
| 1SL8A | 228 | 19 | 0.48 | -1.44 | 24 | 0.40 | -1.07 | 3 | 0.70 | -2.21 | 10 | 0.37 | -1.87 | 2 | 0.68 | -2.10 | 0.05 |
| 1T1VA | 245 | 18 | 0.72 | -1.69 | 22 | 0.10 | -1.09 | 17 | 0.51 | -1.50 | 5 | 0.58 | -2.62 | 5 | 0.67 | -2.37 | 0.00 |
| 1T6UA | 204 | 37 | 0.38 | -1.05 | 62 | 0.06 | -0.64 | 91 | 0.28 | -0.30 | - | - | - | 51 | 0.34 | -0.82 | 0.11 |
| 1TJXA | 263 | 1 | 0.72 | -2.54 | 30 | 0.44 | -1.12 | 10 | 0.68 | -1.36 | 3 | 0.48 | -1.59 | 1 | 0.68 | -1.93 | 0.02 |
| 1TQHA | 226 | 5 | 0.67 | -2.31 | 79 | 0.18 | -0.40 | 1 | 0.74 | -2.71 | 1 | 0.43 | -2.79 | 1 | 0.67 | -2.78 | 0.07 |
| 1TR0A | 223 | 1 | 0.68 | -3.26 | 29 | 0.39 | -1.12 | 9 | 0.51 | -1.71 | 1 | 0.42 | -5.21 | 1 | 0.64 | -3.76 | 0.01 |
| 1U5HA | 223 | 3 | 0.66 | -2.68 | 23 | 0.42 | -1.19 | 10 | 0.62 | -1.58 | 1 | 0.47 | -2.55 | 2 | 0.70 | -2.57 | 0.04 |
| 1UGXA | 262 | 1 | 0.75 | -3.02 | 146 | 0.07 | 0.19 | 1 | 0.68 | -2.87 | 1 | 0.56 | -2.72 | 1 | 0.69 | -2.96 | 0.00 |
| 1WOUA | 249 | 2 | 0.71 | -2.79 | 83 | 0.11 | -0.49 | 1 | 0.78 | -2.61 | 1 | 0.44 | -3.05 | 1 | 0.70 | -2.99 | 0.03 |
| 1X8QA | 258 | 1 | 0.72 | -3.00 | 20 | 0.46 | -1.33 | 3 | 0.74 | -1.79 | 9 | 0.56 | -1.18 | 1 | 0.74 | -2.06 | 0.05 |
| 1XCLA | 241 | 4 | 0.72 | -2.48 | 94 | 0.20 | -0.35 | 1 | 0.68 | -2.61 | 2 | 0.47 | -2.12 | 1 | 0.70 | -2.66 | 0.14 |
| 1XWVA | 263 | 1 | 0.71 | -4.08 | 32 | 0.42 | -1.10 | 1 | 0.66 | -3.72 | 1 | 0.53 | -3.13 | 1 | 0.68 | -3.72 | 0.00 |
| 1YKUA | 189 | 15 | 0.50 | -1.61 | 29 | 0.21 | -1.03 | 3 | 0.60 | -2.43 | - | - | - | 4 | 0.58 | -2.26 | 0.21 |
| 2AVDA | 258 | 4 | 0.62 | -2.19 | 39 | 0.44 | -0.99 | 3 | 0.72 | -2.10 | 1 | 0.36 | -2.38 | 1 | 0.68 | -2.51 | 0.03 |
| 2AZJA | 241 | 15 | 0.63 | -1.69 | 148 | -0.07 | 0.30 | 2 | 0.65 | -2.36 | - | - | - | 3 | 0.67 | -2.20 | 0.03 |
| 2BKFA | 250 | 3 | 0.70 | -2.93 | 52 | 0.23 | -0.81 | 4 | 0.53 | -2.51 | 1 | 0.39 | -5.62 | 1 | 0.61 | -3.93 | 0.03 |
| 2C2QA | 257 | 2 | 0.61 | -2.39 | 64 | 0.19 | -0.77 | 10 | 0.65 | -1.72 | 5 | 0.37 | -1.75 | 2 | 0.58 | -2.11 | 0.08 |
| 2CM4A | 253 | 2 | 0.68 | -2.45 | 35 | 0.37 | -1.13 | 15 | 0.62 | -1.41 | 14 | 0.46 | -1.13 | 1 | 0.70 | -2.02 | 0.02 |
| 2DSKA | 245 | 1 | 0.72 | -2.40 | 27 | 0.55 | -1.12 | 5 | 0.74 | -1.82 | 2 | 0.44 | -2.50 | 1 | 0.73 | -2.40 | 0.12 |
| 2E56A | 260 | 1 | 0.77 | -3.20 | 38 | 0.08 | -1.01 | 1 | 0.69 | -3.44 | 1 | 0.65 | -3.06 | 1 | 0.73 | -3.50 | 0.04 |
| 2GKGA | 242 | 4 | 0.62 | -2.25 | 34 | 0.34 | -1.02 | 7 | 0.72 | -1.66 | 1 | 0.13 | -2.82 | 1 | 0.58 | -2.64 | 0.00 |
| 2GTRA | 231 | 1 | 0.52 | -2.49 | 91 | 0.33 | -0.35 | 5 | 0.48 | -1.93 | 2 | 0.28 | -2.62 | 1 | 0.50 | -2.48 | 0.04 |
| 2H98A | 253 | 1 | 0.75 | -2.74 | 39 | 0.33 | -0.89 | 1 | 0.70 | -3.56 | 2 | 0.54 | -2.10 | 1 | 0.73 | -3.05 | 0.03 |
| 2HY5C | 237 | 1 | 0.62 | -3.20 | 61 | 0.29 | -0.70 | 3 | 0.61 | -2.01 | 1 | 0.39 | -4.38 | 1 | 0.61 | -3.31 | 0.01 |
| 2HYKA | 268 | 1 | 0.73 | -2.55 | 29 | 0.43 | -1.18 | 31 | 0.57 | -1.06 | 1 | 0.64 | -2.72 | 1 | 0.71 | -2.41 | 0.01 |
| 2J9CA | 237 | 6 | 0.56 | -1.76 | 16 | 0.44 | -1.30 | 8 | 0.34 | -1.39 | 2 | 0.35 | -2.53 | 2 | 0.52 | -2.31 | 0.00 |
| 2NZ7A | 217 | 76 | 0.38 | -0.37 | 45 | 0.12 | -0.81 | 90 | 0.30 | -0.30 | - | - | - | 66 | 0.36 | -0.63 | 0.21 |
| 2O6SA | 261 | 2 | 0.72 | -2.53 | 39 | 0.42 | -1.08 | 10 | 0.77 | -1.77 | 1 | 0.45 | -1.86 | 1 | 0.70 | -2.29 | 0.01 |
| 2O70A | 229 | 40 | 0.53 | -0.86 | 59 | 0.18 | -0.69 | 14 | 0.71 | -1.71 | - | - | - | 19 | 0.69 | -1.53 | 0.22 |
| 2OZNA | 265 | 1 | 0.56 | -3.36 | 21 | 0.32 | -1.34 | 4 | 0.54 | -1.38 | 1 | 0.54 | -3.75 | 1 | 0.58 | -3.13 | 0.05 |
| 2PQRA | 233 | 27 | 0.39 | -1.26 | 77 | 0.12 | -0.57 | 6 | 0.48 | -1.60 | - | - | - | 10 | 0.43 | -1.48 | 0.00 |
| 2QWXA | 254 | 2 | 0.54 | -2.68 | 69 | 0.37 | -0.65 | 1 | 0.57 | -2.78 | 1 | 0.31 | -2.68 | 1 | 0.56 | -2.92 | 0.09 |
| 2RINA | 230 | 2 | 0.69 | -2.46 | 194 | -0.26 | 1.03 | 1 | 0.65 | -2.51 | 5 | 0.48 | -1.92 | 1 | 0.63 | -2.38 | 0.19 |
| 2RJ2A | 264 | 2 | 0.63 | -2.62 | 84 | 0.30 | -0.45 | 3 | 0.61 | -2.26 | 4 | 0.47 | -1.80 | 1 | 0.61 | -2.24 | 0.02 |
| 2V2PA | 194 | 31 | 0.56 | -0.98 | 83 | 0.16 | -0.28 | 3 | 0.73 | -1.58 | - | - | - | 8 | 0.69 | -1.41 | 0.06 |
| 2VA0A | 245 | 5 | 0.67 | -2.56 | 75 | 0.06 | -0.62 | 2 | 0.71 | -2.87 | 3 | 0.44 | -1.80 | 3 | 0.67 | -2.81 | 0.01 |
| 2VPVA | 256 | 3 | 0.68 | -2.27 | 154 | 0.02 | 0.24 | 4 | 0.63 | -2.15 | 10 | 0.58 | -1.89 | 1 | 0.64 | -2.07 | 0.07 |
| 2VUHB | 228 | 3 | 0.60 | -2.04 | 83 | 0.16 | -0.40 | 2 | 0.67 | -2.05 | 3 | 0.30 | -2.74 | 1 | 0.60 | -2.46 | 0.03 |
| 2VWSA | 242 | 6 | 0.71 | -1.95 | 55 | 0.19 | -0.77 | 3 | 0.68 | -1.85 | 1 | 0.47 | -2.66 | 1 | 0.69 | -2.45 | 0.09 |
| 2W68A | 268 | 1 | 0.74 | -2.73 | 74 | 0.27 | -0.62 | 51 | 0.59 | -0.90 | 1 | 0.73 | -3.83 | 1 | 0.77 | -3.02 | 0.02 |
| 2WBXA | 269 | 1 | 0.69 | -3.12 | 55 | 0.44 | -0.88 | 1 | 0.65 | -2.83 | 3 | 0.41 | -2.74 | 1 | 0.64 | -2.93 | 0.02 |
| 2WLGA | 255 | 1 | 0.74 | -1.94 | 158 | -0.10 | 0.27 | 9 | 0.73 | -1.68 | 1 | 0.51 | -1.94 | 1 | 0.63 | -2.06 | 0.00 |
| 2XCBA | 220 | 46 | 0.49 | -0.74 | 51 | 0.06 | -0.71 | 42 | 0.59 | -0.91 | - | - | - | 33 | 0.52 | -1.11 | 0.08 |
| 2YZTA | 249 | 53 | 0.41 | -0.84 | 4 | 0.44 | -1.76 | 23 | 0.48 | -1.52 | 11 | 0.16 | -0.98 | 2 | 0.45 | -1.62 | 0.05 |
| 2ZDPA | 233 | 1 | 0.48 | -2.57 | 13 | 0.44 | -1.28 | 26 | 0.33 | -1.18 | 1 | 0.27 | -3.67 | 1 | 0.48 | -3.24 | 0.01 |
| 2ZTBA | 244 | 1 | 0.69 | -3.75 | 41 | 0.27 | -0.97 | 1 | 0.56 | -3.32 | 1 | 0.64 | -2.72 | 1 | 0.68 | -3.37 | 0.00 |
| 3ACHA | 266 | 1 | 0.77 | -2.74 | 40 | 0.29 | -0.91 | 9 | 0.76 | -1.90 | 1 | 0.60 | -2.07 | 1 | 0.72 | -2.27 | 0.01 |
| 3BL2A | 217 | 56 | 0.67 | -0.71 | 38 | 0.12 | -0.94 | 11 | 0.81 | -1.50 | - | - | - | 15 | 0.76 | -1.48 | 0.18 |
| 3BODA | 270 | 1 | 0.77 | -3.02 | 20 | 0.37 | -1.25 | 1 | 0.59 | -2.24 | 1 | 0.71 | -2.62 | 1 | 0.76 | -2.85 | 0.01 |
| 3BQAA | 246 | 4 | 0.68 | -2.23 | 51 | 0.30 | -0.83 | 2 | 0.62 | -2.45 | 3 | 0.53 | -2.00 | 1 | 0.64 | -2.33 | 0.02 |
| 3DCMX | 257 | 1 | 0.70 | -2.79 | 151 | 0.10 | 0.14 | 1 | 0.73 | -2.84 | 2 | 0.47 | -2.65 | 1 | 0.71 | -2.95 | 0.01 |
| 3F2EA | 231 | 41 | 0.50 | -0.99 | 50 | 0.35 | -0.76 | 3 | 0.68 | -1.66 | - | - | - | 12 | 0.65 | -1.46 | 0.09 |
| 3FB5C | 203 | 15 | 0.42 | -1.49 | 98 | 0.15 | -0.01 | 16 | 0.47 | -1.43 | - | - | - | 19 | 0.49 | -1.38 | 0.03 |
| 3FVVA | 246 | 1 | 0.72 | -2.49 | 42 | 0.32 | -0.92 | 1 | 0.77 | -2.17 | 1 | 0.35 | -4.12 | 1 | 0.74 | -2.93 | 0.02 |
| 3GA4A | 248 | 1 | 0.68 | -2.32 | 25 | 0.42 | -1.44 | 1 | 0.73 | -2.34 | 1 | 0.34 | -1.50 | 1 | 0.68 | -2.36 | 0.01 |
| 3HFOA | 236 | 9 | 0.77 | -1.86 | 48 | -0.03 | -0.84 | 11 | 0.70 | -2.02 | 3 | 0.59 | -2.46 | 4 | 0.71 | -2.44 | 0.08 |
| 3IDWA | 208 | 30 | 0.63 | -1.10 | 35 | 0.18 | -0.96 | 18 | 0.67 | -1.36 | - | - | - | 12 | 0.65 | -1.52 | 0.12 |
| 3JXSA | 263 | 1 | 0.77 | -2.68 | 20 | 0.36 | -1.31 | 3 | 0.68 | -2.18 | 1 | 0.64 | -2.10 | 1 | 0.74 | -2.48 | 0.01 |
| 3KZQA | 234 | 4 | 0.71 | -2.36 | 30 | 0.39 | -1.07 | 1 | 0.74 | -2.80 | 1 | 0.40 | -4.04 | 1 | 0.75 | -3.16 | 0.08 |
| 3LCCA | 251 | 1 | 0.71 | -2.17 | 76 | 0.25 | -0.53 | 32 | 0.68 | -1.13 | 2 | 0.41 | -2.20 | 1 | 0.68 | -2.05 | 0.05 |
| 3LLJA | 252 | 3 | 0.72 | -1.91 | 23 | 0.40 | -1.30 | 11 | 0.65 | -1.56 | 5 | 0.44 | -2.18 | 1 | 0.71 | -2.12 | 0.03 |
| 3MD1A | 250 | 3 | 0.68 | -2.62 | 24 | 0.36 | -1.23 | 2 | 0.66 | -2.39 | 2 | 0.27 | -2.63 | 2 | 0.66 | -2.89 | 0.00 |
| 3MMHA | 216 | 10 | 0.52 | -1.74 | 22 | 0.31 | -1.15 | 3 | 0.67 | -2.24 | 6 | 0.30 | -1.13 | 2 | 0.64 | -2.19 | 0.01 |
| Summary |  |  |  |  |  |  |  |  |  |  |  |  |  |  |  |  |  |
| Average | 242.74 | 9.40 | 0.64 | -2.28 | 58.61 | 0.25 | -0.77 | 8.83 | 0.63 | -2.08 | 2.85 | 0.44 | -2.51 | 4.75 | 0.64 | -2.44 | 0.05 |
